# Predicting antipsychotic-induced weight gain in first episode psychosis – A field-wide systematic review and meta-analysis of non-genetic prognostic factors

**DOI:** 10.1192/j.eurpsy.2023.2417

**Published:** 2023-06-06

**Authors:** Ita Fitzgerald, Laura J. Sahm, Amy Byrne, Jean O’Connell, Joie Ensor, Ciara Ní Dhubhlaing, Sarah O’Dwyer, Erin K. Crowley

**Affiliations:** 1 Pharmacy Department, St Patrick’s Mental Health Services, Dublin, Ireland; 2 School of Pharmacy, University College Cork, Cork, Ireland; 3 Pharmacy Department, Mercy University Hospital, Cork, Ireland; 4 Pharmacy Department, Connolly Hospital, Dublin, Ireland; 5 Endocrinology Department, St Columcille’s Hospital, Dublin, Ireland; 6 St Vincent’s University Hospital Group, Dublin, Ireland; 7 Institute of Applied Health Research, College of Medical and Dental Sciences, University of Birmingham, Birmingham, UK; 8 College of Mental Health Pharmacy, Burgess Hill, UK; 9 Department of Medicine, St Patrick’s Mental Health Services, Dublin, Ireland

**Keywords:** Antipsychotic-induced weight gain, Metabolic side effects, Antipsychotics, Risk factors, Prediction

## Abstract

**Background:**

Whether non-genetic prognostic factors significantly influence the variable prognosis of antipsychotic-induced weight gain (AIWG) has not yet been systematically explored.

**Methods:**

Searches for both randomized and non-randomized studies were undertaken using four electronic databases, two trial registers, and via supplemental searching methods. Unadjusted and adjusted estimates were extracted. Meta-analyses were undertaken using a random-effects generic inverse model. Risk of bias and quality assessments were undertaken using Quality in Prognosis Studies (QUIPS) and Grading of Recommendations Assessment, Development and Evaluation (GRADE), respectively.

**Results:**

Seventy-two prognostic factors were assessed across 27 studies involving 4426 participants. Only age, baseline body mass index (BMI), and sex were suitable for meta-analysis. Age (b=−0.044, 95%CI −0.157–0.069), sex (b=0.236, 95%CI −0.086–0.558), and baseline BMI (b=−0.013 95%CI −0.225–0.200) were associated with nonsignificant effects on AIWG prognosis. The highest quality GRADE rating was moderate in support of age, trend of early BMI increase, antipsychotic treatment response, unemployment, and antipsychotic plasma concentration. Trend of early BMI increase was identified as the most clinically significant prognostic factor influencing long-term AIWG prognosis.

**Conclusions:**

The strong prognostic information provided by BMI trend change within 12 weeks of antipsychotic initiation should be included within AIWG management guidance to highlight those at highest risk of worse long-term prognosis. Antipsychotic switching and resource-intensive lifestyle interventions should be targeted toward this cohort. Our results challenge previous research that several clinical variables significantly influence AIWG prognosis. We provide the first mapping and statistical synthesis of studies examining non-genetic prognostic factors of AIWG and highlight practice, policy, and research implications.

## Introduction

Managing antipsychotic-induced weight gain (AIWG) is challenging for patients, clinicians, and policy makers alike. This is partly due to extensive interindividual variability in anthropometric outcomes following antipsychotic commencement. Although antipsychotic choice is an established differentiator of risk [1], genetic and nongenetic prognostic factors have also been studied for their influence on AIWG prognosis [2–4]. A systematic review of pharmacogenomic associations of AIWG concluded that effect sizes of individual gene variants were too small to fulfill the promise of personalized medicine, and that future studies should explore the effects of combining genetic markers alongside clinical variables to improve prediction [3]. However, no similar review of non-genetic prognostic factors has been undertaken to inform such work. Though often ignored amidst the drive for more complex genetic measures, many simple and routinely collected patient characteristics have been shown to influence the prognosis of medication side effects [5, 6].

A range of pre-antipsychotic inititation biological (e.g., thyroid functioning, insulin resistance), clinical (e.g., positive and negative symptom burden), and sociodemographic (e.g., age, gender) variables have been evaluated for their influence on AIWG trajectory [7–11]. In the absence of a systematic appraisal of this research, the value of non-genetic prognostic factors in influencing AIWG prognosis is currently unknown. This includes the number of factors studied and the clinical utility and reliability of reported prognostic associations, for example, in influencing the stratified use of preventative AIWG interventions. Whether current research supports the use of prognostic factors to identify those at the highest risk of AIWG before or upon starting antipsychotic treatment is a question of significant importance to both practitioners and patients given the limited availability of either non-pharmacological or pharmacological management options to reverse AIWG[12, 13]. Whether the value of current non-genetic prognostic factor research is primarily in informing future research, for example, prognostic model development, needs to be addressed. Consequently, non-genetic prognostic factors represent a potentially underexploited resource within AIWG prognostication.

### Objectives

The aim of this review was to identify, synthesize, and appraise research evaluating non-genetic prognostic factors and their association with anthropometric outcomes following antipsychotic initiation. The research question addressed was as follows*:* Among antipsychotic-naïve adults with a first episode of psychosis, are there non-genetic prognostic factors that reliably influence weight and associated outcomes following antipsychotic commencement? Review objectives were as follows:

- Identify what non-genetic prognostic factors have been investigated for their role in AIWG prognosis.
- Determine the direction, strength, and quality of all prognostic factor-outcome associations.
- Explore the clinical utility of any significant and reliable prognostic factor-outcome associations.

For the purposes of this review, a non-genetic prognostic factor-outcome association is defined as any association that does not include the study of a gene variant and its relationship with AIWG prognosis. This includes, but is not limited to, measurement of clinical, sociodemographic, or biological variables and their potential role as prognostic factors.

## Methods

The protocol was registered on PROSPERO (CRD42021258148) and published separately [14]. A detailed outline of review methods is contained within the protocol and a brief description only is provided here. Protocol deviations and a copy of the completed Preferred Reporting Items for Systematic Reviews and Meta-Analyses (PRISMA) statement for this review are contained in the Supplementary Material [15].

### Eligibility criteria

A summary of the modified PICOTS approach recommended for systematic reviews of prognostic factors applied to this review is outlined in Table 1 [16].

**Table 1.** Modified PICOTS criteria applied to this review

Item	Definition
Population	Adult participants (≥16 years of age) diagnosed with a first episode of psychosis in the context of: Brief psychotic disorder Schizophrenia and associated phenotypes, including schizoaffective disorder Delusional disorder Diagnosis is made in accordance with standardized clinical criteria (i.e., DSM-V/ICD-10) Participants must be antipsychotic-naïve. For the purposes of this review, aantipsychotic-naïve is defined as: ≤6 weeks lifetime antipsychotic exposure 0–2 weeks exposure before trial enrolment Never received a long-acting injectable form of antipsychotic We also accepted studies where most participants (≥80%) included met this criterion
Index prognostic factors	Any non-genetic, including clinical (e.g., positive/negative symptomology), sociodemographic (age, sex, socioeconomic status), or biological (e.g., baseline weight, blood markers) prognostic factor measured upon or immediately before antipsychotic initiation and examined prospectively for an association with change in a subsequent anthropometric outcome(s). We also accepted studies that measured a prognostic factor with an initial baseline and subsequent repeat assessment post antipsychotic initiation but within the acute treatment phase that is, change over time was assessed, given the relevance of early change in such variables to the review question.
Comparator prognostic factors	Not applicable
Outcomes	Primary outcomes Relationship between one or more non-genetic prognostic factor and mean change in weight (kg) following antipsychotic commencement Relationship between one or more non-genetic prognostic factor and mean change in body mass index (BMI) following antipsychotic commencement Relationship between one or more non-genetic prognostic factor and likelihood of experiencing clinically significant weight gain following antipsychotic commencement. Clinically significant weight gain is most commonly defined as a ≥ 7% increase in body weight [14], but we accepted studies where this outcome is defined as a ≥ 5% increase Secondary outcomes: Relationship between one or more non-genetic prognostic factor and mean change in waist circumference (cm) following antipsychotic commencement
Timing	No restrictions
Setting	No restrictions

We included both randomized controlled trials (RCTs) and prospective non-randomized studies (NRS) that had a clear inception point. We excluded retrospective or cross-sectional studies to increase evidence certainty [17]. We accepted studies where the prognostic factor effect size was unadjusted or adjusted for other known prognostic factors. We excluded studies that looked solely at antipsychotic class/subclass or gut microbiome variants as potential prognostic factors. We included studies that were classified as exploratory or confirmatory in design. Confirmatory studies are designed to test the independence of a prognostic factor association and thus, provide more conclusive evidence compared to exploratory studies [16]. We classified studies as exploratory or confirmatory according to the author’s objectives and approach to study design and analysis. Strict inclusion criteria were used to limit heterogeneity across studies and facilitate a more meaningful interpretation of synthesized results.

### Search strategy

We conducted focused and broad electronic searches using indexed and free-text words and phrases relating to prognosis, adults with psychosis, and antipsychotic-induced anthropometric changes. PubMed, CENTRAL, PsycINFO, and Embase were initially searched from inception until November 30, 2021. Reference searching, forward citation searching, searching of trial registers, and contacting content experts were undertaken between January 21 and September 30, 2022. The electronic database search was repeated before review completion to include articles published until May 7, 2023 to ensure newly published research was included in the review. Non-English language studies and grey literature were excluded.

### Study selection

Rayyan (rayyan.ai) was used to screen the title and abstracts of all electronic searches. A pre-tested Microsoft Excel sheet was used for studies identified through other sources. Two reviewers independently screened all titles and abstracts (IF, LS, AB, and EC). Disagreements were resolved via consensus and discussion with a third independent reviewer, where required. Study authors were contacted to clarify queries on study conduct or design. The same process was repeated for all full-text articles retrieved. Where multiple studies appearing to use the same or overlapping participant data were identified, we classified the primary study as the publication presenting the most relevant or comprehensive data for our review question.

### Data extraction and management

Data extraction was undertaken using a version of the Checklist for Critical Appraisal and Data Extraction for Systematic Reviews of Prediction Modeling Studies – Modified for Prognostic Factor Studies (CHARMS-PF) [16]. The modified checklist can be found in the study protocol [14]. We extracted all unadjusted and adjusted measures of association and variance estimates from each study. In the case of continuous outcomes, we extracted beta-coefficients and their standard errors (SE). We gave preference to extracting unstandardized beta coefficients (referred to as “b”) to facilitate result interpretation. Where unstandardized coefficients were unretrievable, the standardized beta coefficient (β) was extracted. Only unstandardized coefficients were eligible for meta-analysis. For the outcome risk of clinically significant weight gain (CSWG), we extracted odds ratios (OR) and SE. If these estimates were unavailable, we attempted to recover them using alternative available information provided. Before conversions were undertaken, we contacted authors to request missing results.

### Risk of bias

Risk of bias was assessed independently by pairs of review authors (IF, EC, LS, JOC, and CNiD) using the Quality in Prognosis Studies (QUIPS) tool [18]. Each domain was judged as being at high, moderate, or low risk of bias. An overall rating of bias to a given study is not recommended when using QUIPS [18]. Disagreements between reviewers were resolved via consensus and recourse to a third author, where necessary. Attempts were made to contact study authors for information required to accurately complete assessments.

### Data synthesis

Meta-analysis was conducted when usable data were available reporting the prognostic association between a factor and outcome in ≥ 3 studies deemed sufficiently homogenous. Only adequately adjusted results were considered suitable for meta-analysis [16]. We defined a minimum set of adjustment factors based on existing evidence of their association with weight trajectory in the general population (age, sex, ethnicity) [19], and known influence of antipsychotic prescription on weight prognosis [1]. We conducted all analyses via STATA (StataCorp version 17) with a random-effects generic inverse variance meta-analysis model. Restricted maximum likelihood estimation (REML) was used to fit all analyses, with 95% confidence intervals (CI) derived using the Hartung–Knapp Sidik–Jonkman (HKSJ) approach, to account for uncertainty in estimated variances [20]. Where it was not appropriate to combine studies quantitatively, results were assessed qualitatively. Although our protocol outlined how we would assess for publication bias quantitatively and potential subgroup analyses, in both cases insufficient study numbers prohibited this.

### Certainty of evidence

Evidence quality of each prognostic factor-outcome association was assessed using a Grading of Recommendations Assessment, Development and Evaluation (GRADE) approach modified for prognostic factor research [21]. A quality rating in support of each prognostic factor-outcome association assessed across studies was assigned as high, moderate, low, or very low. Evidence quality was downgraded according to early phase of investigation, study limitations, inconsistency, indirectness, imprecision, and publication bias. Publication bias was assumed in the case of all factors unless a similar association between the factor and outcome had been repetitively assessed in independent studies. Evidence quality could be upgraded where evidence of a moderate–large effect size or exposure-response gradient was consistently demonstrated [21]. GRADE assessments were initially conducted by IF and reviewed independently by a second author (EC and LS). Disagreements were resolved via discussion and recourse to a third reviewer, where necessary.

## Results

### Study selection

**Figure 1.**
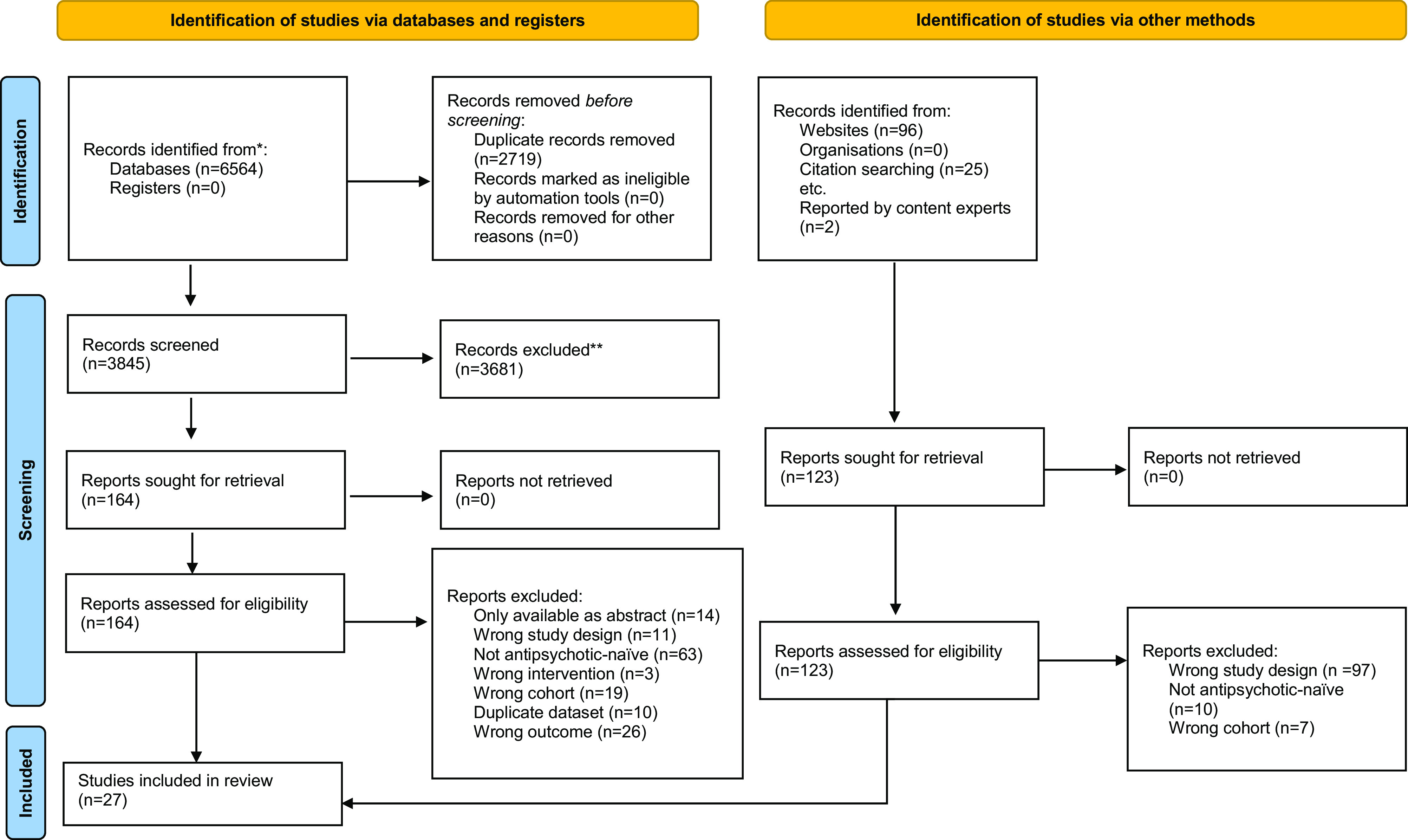
PRISMA flow diagram of study selection process.

After duplicate removal, database searching yielded 3845 articles for the title and abstract screening. Of 164 eligible for full-text review, 18 met the inclusion criteria. Of 123 assessed, a further 9 eligible studies were identified through other search methods. Studies involving apparent duplicate populations were identified in 10 cases. Additionally, in a small number of studies there was evidence of potentially overlapping populations, without clearly highlighting the availability of separate publications elsewhere. Twenty-seven studies were identified for final inclusion. Figure 1 contains a PRISMA flow diagram of the study selection process.

### Study characteristics

Table 2 contains a detailed overview of study characteristics. Seventy-two unique prognostic factors were assessed across studies involving 4426 participants. Across studies, 85% were prospective cohort studies, and the remaining retrospective analyses of RCTs. Study populations comprised both inpatient and outpatient cohorts. 13/27 (n = 3053) studies were classified as exploratory and 14/27 (n = 1373) confirmatory in design. Confirmatory studies generally involved comparatively smaller participant numbers (n < 100), except for two studies [22, 28]. Approximately 37% of studies were conducted in exclusively Chinese populations, although these were primarily exploratory. Change in body mass index (BMI) was the most studied outcome, followed by change in weight, risk of CSWG, and change in waist circumference, respectively. Median follow-up time was 12 weeks (IQR 18) and ranged from 4 weeks to 6 years. 46% of analyses were considered adequately adjusted for covariates. Inadequately adjusted assignment was most commonly due to lack of adjustment for varying antipsychotic prescription [33, 34, 37, 41, 43]. Only one study evaluating birth weight as a prognostic factor assessed for the presence of a non-linear relationship [40].

**Table 2.** Overview of study characteristics of all studies included in the review

First author, year, country,	Population characteristics	Number enrolled	Phase	Primary prognostic factor(s) assessed	Primary outcome(s) assessed
Chen et al. [22]	Age = 27.1 (9.1), 51% male, BMI = 21.4 (3.1)	526	2 – Confirmatory	Response to antipsychotic treatment (positive symptoms, negative symptoms, total psychopathology, general psychopathology)	Weight change over 8 weeks
Pandit et al. [2]	Age = 26.2 (6.08), 71.3% male, BMI = 23.4 (5)	446	1 – Exploratory	Range: age, sex, ethnicity, employment status, primary diagnosis, comorbid MDD, illness severity, previous antipsychotic treatment, inpatient versus outpatient care, baseline body weight	(1) Risk of clinically significant weight gain^a^ over 4 weeks (2) Weight change over 4 weeks
Perez-Iglesias et al. [10]	Age = 27.3 (7.8), 61% male, BMI = 23.2 (3.5)	174	1 – Exploratory	Range: age, sex, ethnicity, employment status, primary diagnosis, comorbid MDD, illness severity, previous antipsychotic treatment, inpatient versus outpatient care, baseline body weight	Weight change over 3,12 and 36 months
Saddichha et al. [9]	Age = 26 (5.5), 52.5% male, BMI = 19.4 (3)	110	1 – Exploratory	Range: antipsychotic prescribed, baseline weight, baseline waist circumference, sex	(1) BMI change, (2) weight change, (3) Risk of clinically significant weight gain^a^ over 6 weeks
Kang et al. [23]	Age = 27.5 (9.73), 37.3% male, BMI = 20.86 (2.20)	51	2 – Confirmatory	Antipsychotic plasma concentration	(1) Weight change, (2) BMI change over 8 weeks
Rasmussen et al. [24]	Age = 26.9 (5.5), 67% male, BMI = 24.2 (4.5)	30	2 – Confirmatory	5HT2A receptor binding capacity	Weight change over 24 weeks
Muntané et al. [25]	Age = 30.63 (9.57), 58% male, BMI = 23.19 (3.81)	381	1 – Exploratory	Range: Age, sex, baseline BMI, antipsychotic prescribed, antipsychotic dose, primary diagnosis, concomitant antidepressant use, BMI increase	BMI change over 12 and 52 weeks
Nielsen et al. [26]	Age = 25 (6), 56% male, BMI = 25.5 (5.5)	69	2 – Confirmatory	Striatal reward activity (right-sided putamen, Left-sided putamen, right ventral striatum, left ventral striatum)	Weight change over 6 weeks
Homan et al. [27]	Age = 21.5 (5.5), 72% male, BMI = 23.6 (4.4)	81	2 – Confirmatory	Striatal volume, Striatal resting-state functional connectivity	Weight change over 12 weeks
Liu et al. [28]	Age = 27.9 (9.3), 55% male, BMI = 21.4 (3.5)	225	2 – Confirmatory	Antioxidant enzymes (Superoxide dismutase (SOD), Catalase (CAT), Glutathione peroxidase (GPx), Malondialdehyde (MDA))	Weight change over 12 weeks
Song et al. [29]	Age = 24.7 (7.5), 53% male, BMI = 20.14 (2.02)	78	2 – Confirmatory	Pro-inflammatory cytokines (IL-1B, IL-6, TNF-alpha)	Risk of clinically significant weight gain^a^ over 24 weeks
Yuan et al. [30]	Age = 23.1 (8.0), 56% male, BMI = 20.54 (2.52)	80	1 – Exploratory	Range: Age, sex, smoking status, disease of illness	(1) Weight change (2) BMI Change over 24 weeks
Lin et al. [31]	Age = 27.60 (8.31), 59% male, BMI = 21.61 (3.91)	22	2 – Confirmatory	Illness severity – positive symptoms	BMI change over mean 6.04 years (2.16)
Medved et al. [32]	Age = 31.07 (7.86), 0% male, BMI = 23.47 (4.43)	94	1 – Exploratory	Range: (antipsychotic prescribed, family history of diabetes mellitus, age, duration of illness, family history of cerebrovascular disorders, family history of obesity, smoking history, illness severity, primary diagnosis)	(1) Waist circumference, (2) BMI change over 12 weeks
Zhang et al. [33]	Age = 26 (8), 50% male, BMI = 22 (3)	117	1 – Exploratory	Range: (baseline BMI, illness severity, sex, age, response to antipsychotic treatment, duration of illness, antipsychotic dose, antipsychotic prescription)	BMI change over 10 weeks
Verma et al. [34]	Age = 29.8 (6.2), 51.89% male, BMI = 21 (3.5)	56	1 – Exploratory	Range: (age, sex, baseline BMI, cumulative antipsychotic exposure, response to antipsychotic treatment – positive symptoms, fasting low density lipoprotein cholesterol (LDL-C), baseline triglycerides, baseline insulin secretion	Risk of clinically significant weight gain^a^ over 24 weeks
Arranz et al. [35]	Age = 24.45 (7.04), 74% male, BMI = 21.75 (3)	38	2 – Confirmatory	Antipsychotic formulation – standard versus disintegrating	(1) Weight change (2) BMI change over 6 weeks
Li et al. [36]	Age = 39.6 (11.5), 44.7% male, BMI = 23.21 (3.84), Diagnosis = FES	296	1 – Exploratory	Range: (age, sex, baseline BMI, systolic blood pressure, fasting triglycerides, fasting high-density lipoprotein cholesterol (HDL-C), fasting LDL-C, total cholesterol, albumin, urea, C-Reactive Protein (CRP), Triiodothyronine (T3), Duration of illness, Waist-hip ratio, Diastolic blood pressure, total protein, fasting plasma glucose, Thyroid stimulating hormone (TSH), Thyroxine (T4))	Weight change over 12 weeks
Chiliza et al. [11]	Age = 24 (6.5), 72% male, BMI = 21.6 (3.9)	133	1 – Exploratory	Range: (age, sex, substance abuse history, history of previous antipsychotic treatment, baseline BMI, baseline HDL-C, baseline LDL-C, fasting glucose, prolactin, baseline triglycerides, total cholesterol	BMI change over 52 weeks
Canal-Rivero et al. [37]	Age = 31.12 (9.76), 55.5% male, BMI = 22.99 (3.88)	596	1 – Exploratory	Range: (age, dietary habits at 3-month follow-up, baseline BMI)	Weight change at 1,2 and 3 years
Huang et al. [38]	Age = 23.5 (no estimate of variance provided), 36.4% male, BMI = 21.3 (1.7)	33	2 – Confirmatory	Early appetite increase	(1) Weight change (2) BMI change over 12 weeks
Luckhoff et al. [39]	Age = 25.8 (6.8), 73% male, BMI = 21.76 (4.04)	90	2 – Confirmatory	Hippocampal subfield volume – anterior + posterior	BMI change over 52 weeks
Zipursky et al. [8]	Age = 23.8 (4.8), 82% male, BMI = 23.8 (4.6)	263	1 – Exploratory	Range: (age, sex, smoking status, primary diagnosis, premorbid functioning, age at illness onset, duration of untreated illness, history of previous antipsychotic treatment)	Risk of clinically significant weight gain over 104 weeks
Garriga et al. [40]	Age = 27.3, 61% male, BMI = 22 (3.1)	23	2 – Confirmatory	Birth weight	Weight change over 16 weeks
Lago et al. [41]	Age = 29.4 (8.5), 62% male, BMI = 22.9 (5.1)	58	2 – Confirmatory	Metabolic markers expressed on peripheral blood mononuclear cells (glucose transporter 1, insulin receptor, fatty acid translocase on CD4+ T cells, CD8+ T cells, CD4–8- T cells, B cells, and monocytes)	BMI change over 12 weeks
Zhou et al. [42]	Age = 26.16 (9.1), 51.2% male, BMI = 21.6 (3.9)	49	2 – Confirmatory	Sex	BMI change over 12 weeks
Vázquez-Bourgon et al. [43]	Age = 29.3 (8.8), 54.5% male, BMI = 23.77 (3.77)	307	1 – Exploratory	Range: (sex, age, duration of untreated psychosis, housing arrangement, ethnicity, smoking status, baseline BMI, baseline waist circumference, positive symptom burden, negative symptom burden)	(1) Weight change (2) BMI change at 52 weeks

Abbreviations: BMI, body mass index; MDD, major depressive disorder.

a Clinically significant weight gain was defined in all studies as a 7% or greater increase in baseline body weight.

### Prognostic factor characteristics

**Figure 2.**
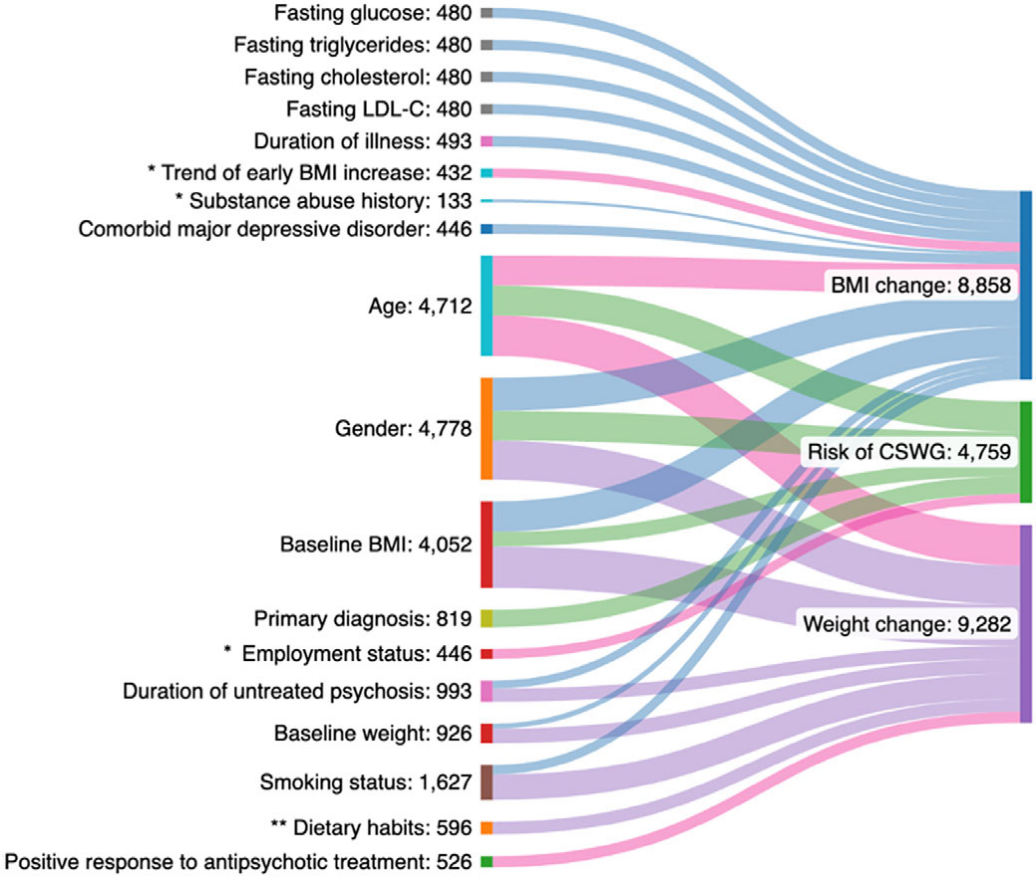
Sankey chart outlining the most frequently studied prognostic factor-outcome associations. Thickness of each line connecting a prognostic factor with an outcome depends on the number of participants across studies examining the association. Each outcome studied is reflected in the diagram by a different color. Lines highlighted in pink specifically indicate moderate quality supporting evidence as per GRADE assessment. * Indicates moderate–large effect size demonstrated. ** Very low-quality supporting evidence for this prognostic factor-outcome association.

A detailed overview of all prognostic factors assessed and reported study estimates are contained within the Supplementary Material. Categories of factors assessed spanned clinical (e.g., psychopathology, comorbid psychiatric diagnoses), neurological (e.g., hippocampal volume, striatal functioning), biological (e.g., antioxidant enzymes, pro-inflammatory cytokines), social (e.g., smoking status), medical (e.g., co-medications), sociodemographic (e.g., age, ethnicity), anthropometric (e.g., pre-antipsychotic weight) and cardiometabolic (e.g., fasting plasma glucose) groupings. The most frequently studied prognostic factor-outcome associations can be found in Figure 2, although in many cases results were not of practical significance. Only moderate-quality results and/or results with potential significant practical impact will be discussed in additional detail in the discussion section. Of the 72 assessed, 65% of prognostic factors were evaluated in a single study. In only two studies was antipsychotic adherence accounted for through design or analysis [11, 39]. One study assessed the impact of diet and lifestyle as a prognostic factor [37]. No analysis adjusted for diet and lifestyle as a covariate.

### Findings from meta-analyses

**Figure 3.**
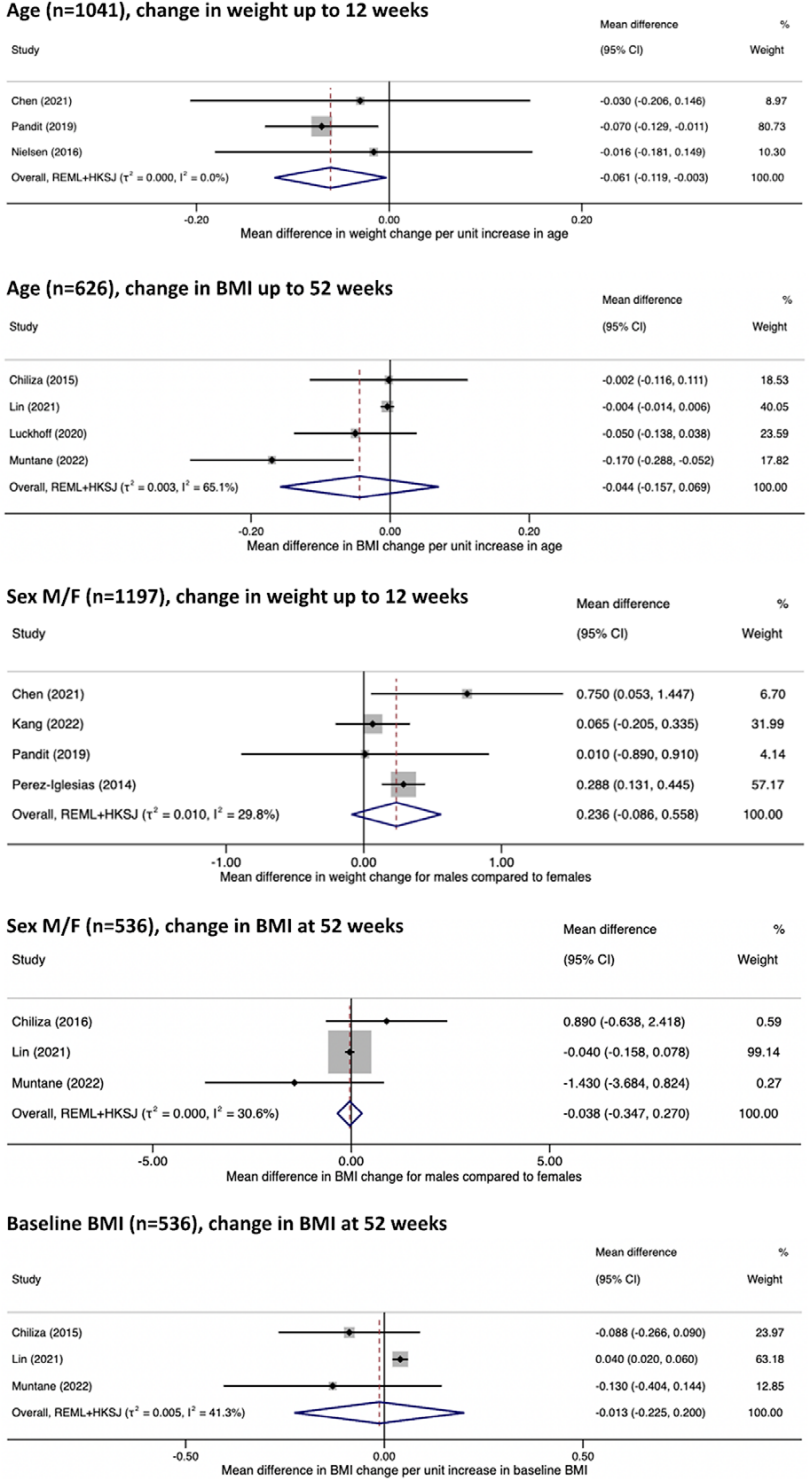
Forest plot of adjusted estimates effects of age, sex, and pre-antipsychotic (baseline) BMI studied for their impact on change in BMI and weight at short- and long-term outcomes.

Prognostic factors eligible for meta-analysis were significantly limited by single study assessments, evaluation of different outcomes (BMI versus weight change) and at varying timelines, and heterogeneity of adjusted covariates. Inclusion in meta-analysis was also hampered by varying measurements of prognostic factors for example, antipsychotic prescription being classified by grouping or by antipsychotic prescribed [9, 10, 22, 25]. Incomplete reporting of results was an additional barrier to meta-analysis [29]. Adjusted estimates of age, sex, and baseline BMI on change in weight and BMI were the only associations judged to be suitable for meta-analysis. The results are displayed in Figure 3. Confidence intervals were wide for all analyses, reflecting large uncertainty due to the small numbers of available study estimates for synthesis. Limited study estimates also led to greater uncertainty in the estimated heterogeneity (tau-squared), and confidence intervals were inflated using the HKSJ method to better account for this uncertainty [44].

### Risk of bias

All studies presented with a moderate risk of bias in at least one domain, and 37% with high risk in at least one domain. The most common section to receive a high risk of bias was statistical analysis and reporting, followed by study attrition and adjustment for other prognostic factors, respectively. Statistical pitfalls noted across studies included insufficient data presentation to assess the adequacy of the analytical approach and inappropriate or misleading modeling strategies for example, reliance on univariable analyses [9, 10, 29, 36]. No study reported an *a priori* sample size calculation. Incomplete or selective reporting of analyses based on presence or absence of statistically significant results was another potential source of bias noted [28, 33, 37, 40]. Inadequate adjustment for covariates limited result interpretation and aggregation [9, 27, 32, 34, 43]. In the case of attrition, sources of potential bias included inappropriate handling of missing data for example, complete case analysis [2, 10, 24, 25, 28, 30, 40, 41]. Reasons for a moderate risk of bias assignment included concerns of selection bias, where in all but one study [9], inadequate description or inappropriate recruitment methods were identified [17]. Unclear measurement properties of prognostic factors were also highlighted in several studies, including how factors were measured and included in analyses [26, 37, 38]. Risk of-bias assessments were often complicated by poor quality reporting of study design, conduct, and particularly analysis methods and associated results. Complete risk of bias results are contained in the Supplementary Material.

### GRADE assessment

Most prognostic factor-outcome associations presented with low-quality supporting evidence. Several had very low-quality evidence ratings. The highest quality rating assigned was moderate and was assigned in the case of:

- Age and BMI change (52 weeks)
- Age and weight change (0–12 weeks)
- Employment status and weight change/risk of CSWG (0–12 weeks)
- Trend of early BMI increase and change in BMI (52 weeks)
- Response to antipsychotic treatment and weight change (0–12 weeks)
- Antipsychotic plasma concentration and change in weight + BMI (0–12 weeks)

Common reasons for downgrading evidence quality included assessment in primarily exploratory studies and risk of bias concerns. Indirectness was identified in several studies where concerns of generalizability were identified [8, 27, 40]. Evidence quality was rated up for employment status and trend of early BMI increase due to reported moderate–large effect sizes across studies. Studies published in the last 5 years generally displayed a higher quality of design, analysis, and reporting [2, 22, 24]. Approximately 93% of confirmatory studies reported at least 1 statistically significant positive result, highly suggestive of publication bias and seen in other similar reviews [45]. Detailed results of all GRADE assessments can be found in the Supplementary Material.

## Discussion

This review is the first systematic collation and exhaustive methodological evaluation of non-genetic prognostic factors studied prospectively for their influence on AIWG prognosis. The review focused on clarifying the current stance of evidence in the area and quantifying the clinical impact and associated reliability of reported prognostic associations. This review followed guidance from the Cochrane Prognosis Methods Group and used several quality design features to produce a transparent evidence summary and increase the strength of review recommendations for practice, policy, and research [16]. We also obtained several datasets where results were not previously published [11, 43]. Although moderate quality evidence was found in support of a small number of factors, for many assessments, defects in study design, analysis, and reporting led to reduced confidence in reported estimates. Certainty in reported prognostic factor-outcome associations was often limited by small sample sizes, brief evaluation periods, and concerns of bias, including publication bias. Taken together, most prognostic factor-outcome associations highlighted in this review require further independent study to confirm results. Although reasons will be discussed further, review conclusions are summarized in Table 3.

**Table 3.** Summary of review conclusions

Category	Conclusion	Practice and policy implications	Research implications
1 – Measurement of prognostic factors evaluated *prior to* antipsychotic initiation and use of results to inform AIWG prognosis	Insufficient evidence exists to recommend routine measurement of any nongenetic prognostic factor *prior* to antipsychotic initiation	Currently insufficient evidence exists to support use of nongenetic prognostic factors to inform AIWG management for example, risk stratified use of interventions to prevent AIWG according to baseline risk	We identified several prognostic factors that should be prioritized for evaluation in future confirmatory studies with extended follow-up. This includes employment status, antipsychotic plasma concentration, and substance misuse history. Only substance misuse history had a protective effect on AIWG prognosis
2 – Clinical variables previously thought to be prognostic	Patient characteristics previously reported to influence AIWG prognosis including age and antipsychotic treatment response [46, 47] likely play a clinically insignificant role in long-term AIWG prognosis. Our analysis did not provide conclusive evidence that sex or baseline BMI significantly impact AIWG prognosis at 1 year		The role of sex and baseline BMI on AIWG prognosis requires further study given low-quality evidence supporting conclusions. Effects of sex in studies with longer-term follow-up are required given increased risk found here in short-term analyses. Adjustment for age in prognostic model development, clinical trial design, or in assessments of independent associations of novel prognostic factors is likely not essential
3 – Prognostic value of the antipsychotic prescription	The antipsychotic prescribed remains the most significant baseline variable in influencing AIWG prognosis [1]. This review did not find conclusive evidence to support antipsychotic dose in meaningfully impacting AIWG prognosis	Significant differences in propensity to cause weight gain among antipsychotics should be accounted for within AIWG management guidelines given the strong prognostic information provided by use of low- versus high-risk agents. The absence of evidence demonstrating a protective effect of lower doses on AIWG prognosis should also be highlighted	Our results underscore the importance of evidence-based antipsychotic prescribing in first-episode psychosis. Research into methods to reduce indiscriminate use of olanzapine and medium-risk antipsychotics in this context is needed [48, 49]. Research is also required as to whether a differentiated approach to management based on antipsychotic prescribed is more effective in managing AIWG compared to the currently endorsed uniform approach, independent of the antipsychotic prescribed
4 – Prognostic value of early BMI increases	Trend of BMI increase within the first 12 weeks of antipsychotic treatment likely provides strong prognostic information regarding extended AIWG prognosis. Those who experience significant weight gain (most commonly defined as a ≥ 5% increase in baseline body weight) early in treatment have a worse long-term prognosis when compared to those who don’t [50].	Among those who experience significant BMI increases early in treatment, current stepwise management algorithms should likely be accelerated and intensified [51–53]. Given the higher likelihood of experiencing absolute benefits and resources required to implement their use, use of intensive and individualized interventions would likely be used more efficiently among this group [12]. Pharmacological treatments, including those shown to be more effective at plateauing versus treating AIWG that is, metformin [53–55] may also be more beneficial among this group	Significant increases in BMI early in treatment likely represent phenotypic expression of those at higher inherent risk of AIWG and may be a more efficient way of measuring genetic correlates of AIWG and targeting management interventions accordingly. Whether use of early BMI trend to inform AIWG management leads to a sustained positive impact on AIWG prognosis requires evaluation. Differential treatment responses to pharmacological and non-pharmacological interventions among those whose trend of initial AIWG is more extensive is also worthy of future research
Additional research recommendations			
5 – Next steps	This review provides a blueprint for next steps in prognostic factor research in AIWG prognosis and has implications for both nongenetic and genetic prognostic research, for example, in developing individualized AIWG prediction models. New studies should build on the results outlined here, including: Prognostic factors deserving of prioritization for further study, and methodological and reporting improvements required to establish evidence-based prognostic assessments, have been highlighted here. Guidelines on both reporting and methodological standards of prognostic factor research have been previously published and should be adhered to in future studies [56, 57] Results should be used by researchers to improve the quality of future studies as well as ensuring evaluation of factors and outcomes similarly to facilitate meaningful comparison and meta-analysis of collective results Whether relationships between prognostic factors and outcomes assessed may have become apparent, or become more clearly elucidated, through assessment for non-linear relationships is an important question that remains unaddressed A lack of study of prognostic factors operating at a broader contextual level was identified, including known determinants of weight for example, diet and lifestyle variables. Future research should consider this and need for assessment of potential prognostic factors at an ecological level for example, area-level social deprivation, healthcare access and physical environment given their relevance to the cohort under investigation		

Prognostic factor-outcome associations with the greatest evidence certainty and results of highest practical importance, and from which conclusions in Table 3 were based on, will now be discussed. Factors with very low-quality supporting evidence will not be discussed. The value of sociodemographic and clinical variables previously thought to be prognostic including age, sex, baseline BMI and response to antipsychotic treatment will also be discussed [46, 58]. Reasons for downgrading quality for each prognostic factor-outcome association and factors evaluated due to their potential role in AIWG etiology are discussed in the Supplementary Material.

### Age

Most studies reported a small average negative effect of age on prognosis of weight increase [22, 26, 28], BMI increase [11, 25, 31, 36, 39, 43], and risk of CSWG [2, 9]. Results were similar in the case of varying follow-up durations [2, 24, 34, 43], antipsychotic prescribed [2, 11, 24], and study setting [2, 11, 25, 34]. A similarly small average negative effect estimate was also seen independent of study quality, including risk of bias rating [2, 34, 43], although study precision in demonstrating a consistent negative effect was greater in better quality and larger studies [24, 43]. Meta-analysis results for change in weight and BMI were similar where reported effect sizes and the upper and lower limits of reported confidence intervals were compatible with no significant effect of age on AIWG prognosis. Evidence quality was moderate for adjusted estimates of BMI and weight change at all timepoints.

### Sex

Sex was assessed in 11 exploratory and one confirmatory study. Results varied across studies, which may have been the result of heterogeneity in study design, particularly length of follow-up, and study quality [2, 11, 34, 42]. In studies assessed qualitatively, nonsignificant differences between sex were largely seen across short- [9, 30, 33, 34, 36] and longer-term studies [8, 43], and in both unadjusted [9, 36] and adjusted analyses [8, 30]. Meta-analysis of short-term studies found males to be at higher risk of AIWG, although the difference in effect size was not practically important. Increased risk among males was not seen in the meta-analysis of longer-term studies, although this analysis was limited by small study numbers and weight largely being assigned to a single study. For both outcomes quality was deemed low. Given the low-quality evidence, conclusions regarding sex’s role on AIWG prognosis are uncertain.

### Early BMI increase

Baseline BMI was the most studied anthropometric measurement assessed for its impact on AIWG prognosis. Meta-analysis of studies with a follow-up of 12 months found no significant effect of baseline BMI on AIWG prognosis. However, evidence quality for baseline BMI as a prognostic factor was low for all outcomes, except for a change in waist circumference, where quality was very low. Thus, like sex, although a significant impact of baseline BMI was not seen here, results are inconclusive. Assessment of early anthropometric changes post antipsychotic initiation demonstrated significantly more prognostic promise. Several studies assessed the impact of early anthropometric changes on weight and BMI prognosis up to 52 weeks. One study (n = 381) assessed the value of 12-week BMI change on final BMI change at 12 months among participants prescribed varying antipsychotics and reported b = 0.89 (95% CI 0.73–1.05, *p* < 0.001) that is, every one-unit BMI increase at 12 weeks predicted an almost identical BMI increase at 1 year. Trend of early BMI increase explained approximately 30% variance in final BMI change – more than age, sex, baseline BMI, antipsychotic prescribed, and dosage combined [25]. In another study (n = 51), early BMI rate of change correlated almost perfectly with BMI and weight change at 8 weeks treatment, r = 0.988 and r = 0.992, *p* < 0.001, respectively [23]. In a 10-year follow-up, percentage weight increase at 12 months treatment had a significant prognostic effect on the odds of experiencing a 20% weight increase at 10 years [43]. Early BMI increase as a prognostic factor was judged to have moderate quality supporting evidence. Confirmatory evidence for the prognostic role of early anthropometric changes was also provided indirectly through other included studies. One study assessed the role of early appetite increase on AIWG prognosis and found that increased appetite at 4 weeks was positively associated with increased weight gain at 12 weeks, b = 0.67 (95% CI 0.31–1.03), *p* = 0.0003 [38]. At 12 weeks, participants with an earlier appetite increase (0–4 versus 4–8 weeks treatment) showed significantly greater weight gain, mean difference = 2.67 kg (95% CI 1.20–4.15), *p* < 0.0001 [38]. Quality for early appetite increase as a prognostic factor was judged to be low. Previous research in non-antipsychotic-naïve cohorts (n = 351) found that 5% weight increase at 1-month treatment predicted weight gain of 15% at 3 months (sensitivity 67%, specificity 88%, *p* = 0.001). Among those who gained <5% at 1 month, average weight gain at 3 months was significantly lower when compared to the ≥5% group, 2.4% versus 8.1%, *p* = 0.0005 [50].

### Antipsychotic dose

Exploratory studies assessing the prognostic value of antipsychotic dose on weight and BMI change were identified [23, 24, 33]. In the only study with numerical results [25], adjusted analysis found insignificant effect sizes of <0.01 on BMI change at 3 (*p* = 0.19) and 12 months (*p* = 0.24). Evidence quality was low and very low for impact on change in BMI and weight, respectively. One confirmatory study (n = 51) in China assessed the role of 4-week olanzapine plasma concentration in influencing weight and BMI change at 8 weeks and reported β = 0.376 (95% CI 0.08–0.67), *p* = 0.013 for weight change and β = 0.354 (95% CI 0.06–0.65), *p* = 0.019 for BMI change, suggesting a potential modest role of antipsychotic plasma concentration as a prognostic factor [23]. Evidence quality was moderate for antipsychotic plasma concentration as a prognostic factor. A previous meta-analysis assessing the impact of antipsychotic dose on weight trajectory found hyperbolic dose curves for most antipsychotics, that is, initial dose-related weight increases, with a subsequent plateau at higher doses. However, for most assessments, average weight differences between higher and lower doses did not differ to a clinically significant extent, although studies were of short duration [59]. A separate review highlighting dose reduction as ineffective in significantly reversing AIWG supports the hypothesis of AIWG being somewhat dose-independent [60], although the absence of linear relationships may mean dose reductions are more effective within certain ranges. Taken together, additional research is required to confirm whether antipsychotic dosing meaningfully impacts AIWG prognosis. Future studies should be designed specifically to test this hypothesis and should assess the prognostic value of plasma concentration rather than the dose to account for varying pharmacokinetics, account for potential trend differences between antipsychotics, evaluate the presence of non-linear relationships and assess dose ranges and timelines reflective of clinical practice.

### Response to antipsychotic treatment

A positive response to antipsychotic treatment has previously been associated with negatively impacting AIWG prognosis, although the clinical significance of the relationship has been unclear [47]. A confirmatory study (n = 529) in China assessed the association between AIWG and antipsychotic response measurements and reported weak correlations between weight gain and reduction in Positive and Negative Syndrome Scale (PANSS) positive, negative, and total subscores at 8 weeks. Adjusted analysis found a clinically insignificant positive association between total PANSS reduction and AIWG, β=0.03 (95% CI 0.01–0.05), *p* = 0.007 [22]. In a separate study (n = 56), percentage change in PANSS positive subscale was not associated with substantial risk of CSWG at 6 months, OR = 1.0 (95% CI 0.9–1.2), *p* = 0.77 [34]. Evidence quality was moderate for weight change and low for all other outcomes. Results here signify that treatment response is not a practically useful prognostic marker, although repeat assessments in additional ethnic groups are required to confirm generalizability of findings.

### Moderate–large effect sizes

We identified a small number of prognostic factors with a reported moderate–large effect size. In one study (n = 446) without serious limitations, unemployment was associated with an increased risk of CSWG at 4 weeks treatment, OR = 2.83 (95% CI 1.50–5.36), *p* = 0.001. A significant impact on weight change was also reported [2]. Evidence quality for unemployment as a prognostic factor was moderate. AIWG is mediated primarily through increased appetite and food cravings [46]. Unemployment may impact AIWG prognosis through increased consumption of higher calorie foods typically more readily consumed by those on lower incomes, as well as reduced access to lifestyle interventions shown to attenuate AIWG [12]. In another study [11], a positive history of substance misuse was associated with a protective effect on BMI increase at 52 weeks treatment, b = −2.25 (95% CI −3.66–(−0.84)), *p* = 0.002. Substance abuse history may be a proxy for other prognostic factors, for example, poor self-care, or may provide supporting evidence for the striatum’s role in AIWG etiology, as previously demonstrated [26, 27]. However, the evidence quality for this prognostic factor was low. Given the practical implications of the reported range of effect sizes for both patients and clinicians, valid mechanisms underlying potential associations, and rates of occurrence in the population of interest, their independent prognostic value deserves further experiment in confirmatory studies.

### Limitations

Review limitations include the exclusion of non-English language papers. Although a previous evidence review has suggested otherwise [61], there is potential that this decision may have introduced bias if statistically or clinically significant results were more likely to have been published in an English language journal. All attempts were made to avoid the inclusion of overlapping participant populations. However, in a minority of cases where clarification was not received, review authors decided on the likelihood that overlapping populations were present. This may mean that some studies were included or excluded inappropriately. As highlighted in similar reviews [40], primary studies may have been missed during searching due to lack of standardized indexing of prognostic factor studies. Exclusion of cross-sectional and retrospective study designs inherently increased evidence certainty underlying prognostic factor-outcome assessments but may have excluded studies that identified candidate factors deserving of further study. Individual participant data meta-analysis may have addressed some of the limitations identified in this review for example, publication bias or inappropriate statistical analyses [16], but was beyond the scope of this review.

## Data Availability

No new datasets were generated via this study. The dataset that was reanalyzed is available here: http://doi.org/10.17632/b2h5gr9m3c.1. Study estimates that were usable and extracted for all relevant outcomes are outlined in the Supplementary material. Data extraction forms can be found in the published study protocol.

## Supplementary material

For supplementary material accompanying this paper visit http://doi.org/10.1192/j.eurpsy.2023.2417. click here to view supplementary material
