# Supplementary material for: Predicting antipsychotic-induced weight gain in first episode psychosis – A field-wide systematic review and meta-analysis of non-genetic prognostic factors
Source: Eur Psychiatry. 2023 Jun 6;66(1):e42. doi: 10.1192/j.eurpsy.2023.2417 (PMC10305761; doi:10.1192/j.eurpsy.2023.2417)
Supplement: Supplementary file 1 [file S0924933823024173sup001.docx]

**Supplementary appendix**

***Predicting antipsychotic-induced weight gain in first episode psychosis – a field-wide systematic review and meta-analysis of non-genetic prognostic factors***

Ita Fitzgerald, Laura J. Sahm, Amy Byrne, Jean O’Connell, Joie Ensor, Ciara Ní Dhubhlaing, Sarah O’Dwyer, Erin K. Crowley

Table of Contents

[Part 1 - List of protocol deviations 2](#_Toc130535657)

[Part 2 - PRISMA checklist 4](#_Toc130535658)

[Part 4 – Overview and categorisation of all prognostic factors studied. 10](#_Toc130535659)

[Part 5 – Usable study estimates from all included papers 11](#_Toc130535660)

[Part 6 – Risk of bias assessment results 23](#_Toc130535661)

[Part 7 - GRADE prognostic factor-outcome assessments 24](#_Toc130535662)

[Section 1 - GRADE tables for all prognostic factor-outcome associations assessed narratively only. 25](#_Toc130535663)

[Table 1 – Prognostic factors and association with risk clinically significant weight gain at all timepoints. 25](#_Toc130535664)

[Table 2 – Prognostic factors and association with change in waist circumference assessed at all timepoints. 28](#_Toc130535665)

[Table 3 – Prognostic factors and association with change in weight at all timepoints. 30](#_Toc130535666)

[Table 4 – Prognostic factors and association with change in BMI at all timepoints. 35](#_Toc130535667)

[Section 2 - GRADE tables for all prognostic factor-outcome associations assessed via adjusted meta-analysis. 39](#_Toc130535668)

[Table 1 – Prognostic factors and association with change in BMI assessed at 52 weeks+ 39](#_Toc130535669)

[Table 2 – Prognostic factors and association with change in weight assessed at 0-12 weeks. 40](#_Toc130535670)

[Part 8 – Novel prognostic factors studied. 43](#_Toc130535671)

# Part 1 - List of protocol deviations

1. Prognostic factors under investigation were initially grouped under ‘biological, clinical and sociodemographic’ in the protocol. In the final paper, this was renamed as ‘non-genetic prognostic factors’ as it was agreed that this better explained the range of factors the review could potentially include.
2. In the protocol we specified that studies for inclusion must measure potential prognostic factors upon or immediately prior to antipsychotic initiation and referred to these as ‘baseline’ measurements. We amended this definition after publication of the protocol to allow inclusion of factors that had a baseline measurement and a follow up measurement early in antipsychotic treatment e.g., trend of early body mass index (BMI) increase, early assessment of response to antipsychotic treatment, and changes in appetite in the weeks following antipsychotic initiation. This was amended due to the relevance of such results to the review question. We also included one study that assessed antipsychotic plasma concentration measurement after steady state had been reached and the associated impact on prognosis.
3. In the protocol we specified that the median or mean dose of antipsychotic prescribed must be included in the study report. We subsequently added dose range to this list.
4. Preferred effect sizes for extraction in the case of continuous outcomes in the protocol were regression coefficients. We subsequently further defined this as a preference for extraction of unstandardized beta coefficients for ease of interpretation.
5. In the protocol we specified that we would calculate a 95% prediction interval for prognostic factors where there were 5 or more estimates available from independent studies suitable for meta-analysis. Study numbers precluded this.
6. We specified in the protocol that meta-analysis would be conducted by grouping studies together where outcomes were measured at similar time points. We subsequently further defined similar timelines this as studies with a follow-up of 0-12 weeks, 12-51 weeks and 52 weeks+. We identified one study that had a follow up time >52 weeks. In the case, relevant study findings were synthesized with studies that had a 52-week follow-up period.
7. In the protocol we specified how publication bias would be assessed quantitatively. Study numbers precluded this.
8. In our protocol, we specified a list of sensitivity and subgroup analyses that would be conducted in the presence of sufficient study numbers. This was not possible due to insufficient study numbers and study heterogeneity.
9. In conducting our searches, we identified one study where a full dataset was provided but analyses reported did not meet our prespecified criteria. The statistician (JE) in our group re-analysed the datasets to adjust adequately for preferred covariates and to report outcomes relevant to our review.
10. In our protocol, we originally planned that all GRADE assessments would be done in duplicate independently. This was changed in our final analysis plan whereby GRADE assessments were completed in full by one author (IF) and their results were reviewed independently by two other authors (LS, EC) until consensus was agreed on all decisions. Furthermore, we subsequently agreed to change initially highlighted GRADE prognostic factor methodology once we discovered primarily continuous outcomes were assessed in eligible studies. Our initial methodology chosen was relevant for prognostic factor research but where outcomes were binary. The approach we subsequently used was by Huguet et al,^1^ and considers similar categories to rate down and rate up evidence quality. In line with this approach,^1^ we also chose to use GRADE tables outlined in the appendix of their 2013 paper rather than GRADEpro web application initially specified in the protocol.

***References***

1 - Huguet A, Hayden JA, Stinson J, McGrath PJ, Chambers CT, Tougas ME, et al. Judging the quality of evidence in reviews of prognostic factor research: adapting the GRADE framework. Syst Rev [Internet]. 2013 Sep 5 [cited 2022 Oct 23];2:71. Available from: <https://www.ncbi.nlm.nih.gov/pmc/articles/PMC3930077/>

| Part 2 - PRISMA checklist | | | |
| --- | --- | --- | --- |
| **Section and Topic** | **Item #** | **Checklist item** | **Location where item is reported** |
| **TITLE** | | |  |
| Title | 1 | Identify the report as a systematic review. | Title |
| **ABSTRACT** | | |  |
| Abstract | 2 | See the PRISMA 2020 for Abstracts checklist. | Adhered to BMJ Mental Health requirements |
| **INTRODUCTION** | | |  |
| Rationale | 3 | Describe the rationale for the review in the context of existing knowledge. | Introduction |
| Objectives | 4 | Provide an explicit statement of the objective(s) or question(s) the review addresses. | Introduction |
| **METHODS** | | |  |
| Eligibility criteria | 5 | Specify the inclusion and exclusion criteria for the review and how studies were grouped for the syntheses. | Table 1 of study paper and reference to published protocol containing detailed methods. |
| Information sources | 6 | Specify all databases, registers, websites, organisations, reference lists and other sources searched or consulted to identify studies. Specify the date when each source was last searched or consulted. | Search strategy section and reference to published protocol containing detailed methods. |
| Search strategy | 7 | Present the full search strategies for all databases, registers and websites, including any filters and limits used. | Not contained in study report. Contained in protocol paper referenced in study report. |
| Selection process | 8 | Specify the methods used to decide whether a study met the inclusion criteria of the review, including how many reviewers screened each record and each report retrieved, whether they worked independently, and if applicable, details of automation tools used in the process. | Study selection section and reference to published protocol containing detailed methods. |
| Data collection process | 9 | Specify the methods used to collect data from reports, including how many reviewers collected data from each report, whether they worked independently, any processes for obtaining or confirming data from study investigators, and if applicable, details of automation tools used in the process. | Data extraction and management section and reference to published protocol containing detailed methods. |
| Data items | 10a | List and define all outcomes for which data were sought. Specify whether all results that were compatible with each outcome domain in each study were sought (e.g. for all measures, time points, analyses), and if not, the methods used to decide which results to collect. | Table 1 of study paper and reference to published protocol containing detailed methods. |
|  | 10b | List and define all other variables for which data were sought (e.g. participant and intervention characteristics, funding sources). Describe any assumptions made about any missing or unclear information. | Data extraction and management section and reference to published protocol containing detailed methods. |
| Study risk of bias assessment | 11 | Specify the methods used to assess risk of bias in the included studies, including details of the tool(s) used, how many reviewers assessed each study and whether they worked independently, and if applicable, details of automation tools used in the process. | Risk of bias section and reference to published protocol containing detailed methods. |
| Effect measures | 12 | Specify for each outcome the effect measure(s) (e.g. risk ratio, mean difference) used in the synthesis or presentation of results. | Data extraction and management section and reference to published protocol containing detailed methods. |
| Synthesis methods | 13a | Describe the processes used to decide which studies were eligible for each synthesis (e.g. tabulating the study intervention characteristics and comparing against the planned groups for each synthesis (item #5)). | Data synthesis section and reference to published protocol containing detailed methods. |
|  | 13b | Describe any methods required to prepare the data for presentation or synthesis, such as handling of missing summary statistics, or data conversions. | Data extraction and management section, data synthesis section and reference to published protocol containing detailed methods. |
|  | 13c | Describe any methods used to tabulate or visually display results of individual studies and syntheses. | Reference to published protocol containing detailed methods. |
|  | 13d | Describe any methods used to synthesize results and provide a rationale for the choice(s). If meta-analysis was performed, describe the model(s), method(s) to identify the presence and extent of statistical heterogeneity, and software package(s) used. | Data synthesis section and reference to published protocol containing detailed methods. |
|  | 13e | Describe any methods used to explore possible causes of heterogeneity among study results (e.g. subgroup analysis, meta-regression). | n/a due to small study numbers. Methods originally outlined in published protocol. |
|  | 13f | Describe any sensitivity analyses conducted to assess robustness of the synthesized results. | n/a due to small study numbers. Methods originally outlined in published protocol. |
| Reporting bias assessment | 14 | Describe any methods used to assess risk of bias due to missing results in a synthesis (arising from reporting biases). | n/a due to small study numbers. Methods originally outlined in published protocol. |
| Certainty assessment | 15 | Describe any methods used to assess certainty (or confidence) in the body of evidence for an outcome. | Certainty of evidence section and reference to published protocol containing detailed methods. |
| **RESULTS** | | |  |
| Study selection | 16a | Describe the results of the search and selection process, from the number of records identified in the search to the number of studies included in the review, ideally using a flow diagram. | Figure 1 |
|  | 16b | Cite studies that might appear to meet the inclusion criteria, but which were excluded, and explain why they were excluded. | Figure 1 |
| Study characteristics | 17 | Cite each included study and present its characteristics. | Table 2 |
| Risk of bias in studies | 18 | Present assessments of risk of bias for each included study. | Risk of bias results section + Part 5 of supplementary appendix. |
| Results of individual studies | 19 | For all outcomes, present, for each study: (a) summary statistics for each group (where appropriate) and (b) an effect estimate and its precision (e.g. confidence/credible interval), ideally using structured tables or plots. | Findings from meta-analysis section and Part 4. |
| Results of syntheses | 20a | For each synthesis, briefly summarise the characteristics and risk of bias among contributing studies. | Each prognostic factor-outcome estimate has GRADE evidence quality rating which accounts for risk of bias ratings for each contributing study. Additionally, for use of QUIPS tool, overall study risk of bias rating not recommended, rating of bias amongst each section preferred. |
|  | 20b | Present results of all statistical syntheses conducted. If meta-analysis was done, present for each the summary estimate and its precision (e.g. confidence/credible interval) and measures of statistical heterogeneity. If comparing groups, describe the direction of the effect. | Findings from meta-analysis section |
|  | 20c | Present results of all investigations of possible causes of heterogeneity among study results. | n/a due to small study numbers. |
|  | 20d | Present results of all sensitivity analyses conducted to assess the robustness of the synthesized results. | n/a due to small study numbers. |
| Reporting biases | 21 | Present assessments of risk of bias due to missing results (arising from reporting biases) for each synthesis assessed. | n/a due to small study numbers. |
| Certainty of evidence | 22 | Present assessments of certainty (or confidence) in the body of evidence for each outcome assessed. | Figure 2 and Part 6 of supplementary appendix. |
| **DISCUSSION** | | |  |
| Discussion | 23a | Provide a general interpretation of the results in the context of other evidence. | Conclusions and implications section and Table 3. |
|  | 23b | Discuss any limitations of the evidence included in the review. | Limitations section |
|  | 23c | Discuss any limitations of the review processes used. | Limitations section |
|  | 23d | Discuss implications of the results for practice, policy, and future research. | Conclusions and implications section and Table 3. |
| **OTHER INFORMATION** | | |  |
| Registration and protocol | 24a | Provide registration information for the review, including register name and registration number, or state that the review was not registered. | Abstract |
|  | 24b | Indicate where the review protocol can be accessed, or state that a protocol was not prepared. | Referenced in study selection and analysis section. |
|  | 24c | Describe and explain any amendments to information provided at registration or in the protocol. | List of protocol deviations are outlined in Part 1 of the supplementary appendix. |
| Support | 25 | Describe sources of financial or non-financial support for the review, and the role of the funders or sponsors in the review. | Funding section. |
| Competing interests | 26 | Declare any competing interests of review authors. | Competing interest section |
| Availability of data, code and other materials | 27 | Report which of the following are publicly available and where they can be found: template data collection forms; data extracted from included studies; data used for all analyses; analytic code; any other materials used in the review. | Data availability statement |

# Part 4 – Overview and categorisation of all prognostic factors studied.


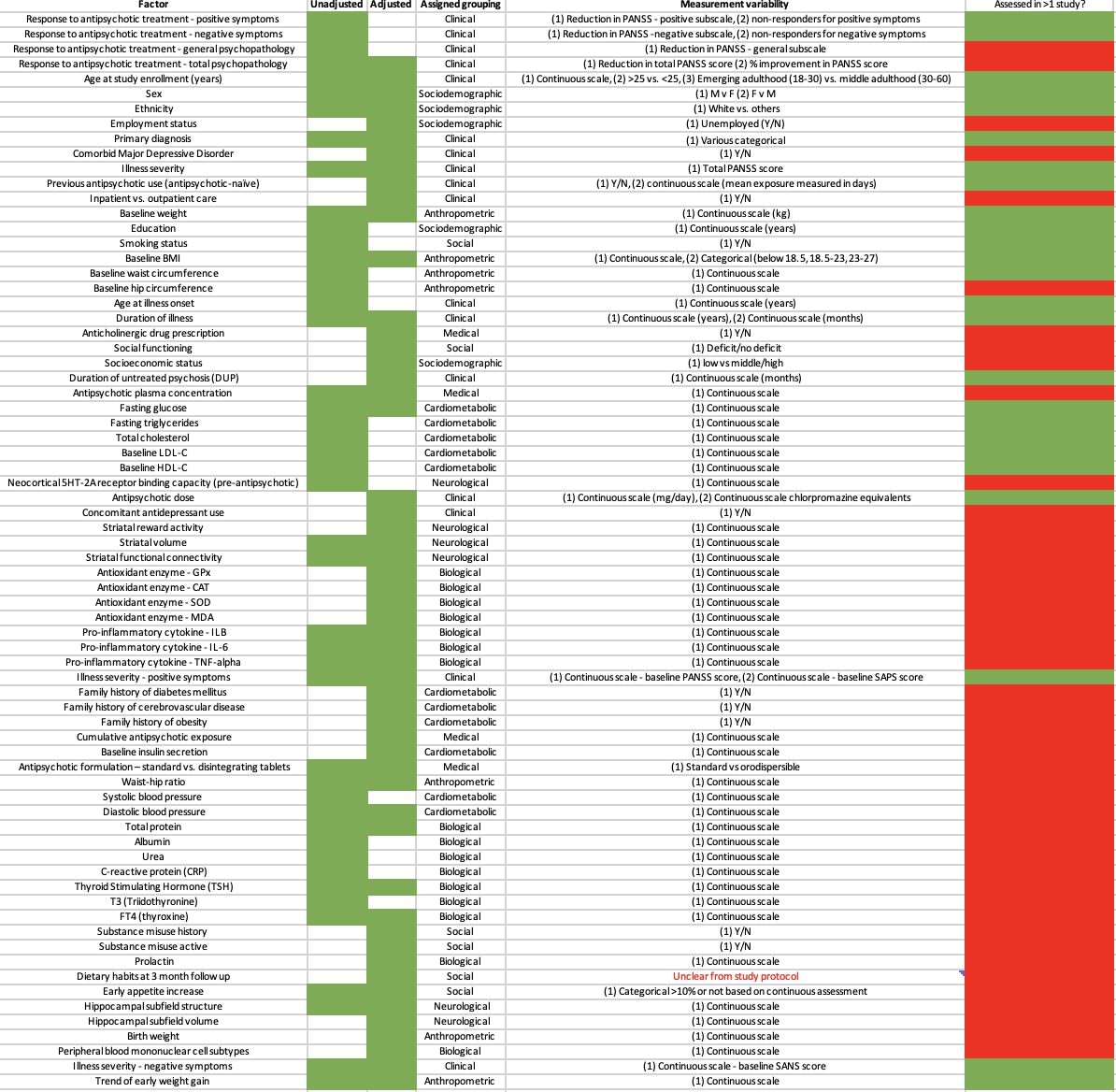


Part 5 – Usable study estimates from all included papers**.**

Only relevant estimates to the review question are included here. For full details, the study paper should be referred to.

| **Study** | **Prognostic factor** | **Outcome** | **Analysis** | **Study estimates + confidence intervals + P value (where available or could be estimated from study report)** |
| --- | --- | --- | --- | --- |
| Chen (2021) | Confirmatory study - Response to antipsychotic treatment   - Reduction in positive symptoms - Reduction in negative symptoms - Reduction in total psychopathology - Reduction in general psychopathology | Change in weight at 8 weeks | Unadjusted = Pearson’s correlation analysis  Adjusted - Multivariable linear regression | **Unadjusted estimates (r) =**  Decrease in PANSS positive = 0.14, p = 0.004  Decrease in PANSS negative = 0.04, p = 0.47  Decrease in PANSS general psychopathology = 0.1  Decrease in PANSS total = 0.18, p <0.001  **Linear regression results (β**) **=**  Age (years) = - 0.06 (95% CI -0.44 to 0.31), p = 0.73  Gender (F/M) = - 0.75 (95% CI -1.44 to -0.05), p = 0.035  Education (years) = 0.03 (95% CI -0.26 to 0.04), p = 0.45  Smoking (Y/N) = - 0.04 (95% CI -1.46 to 1.38), p = 0.96  Baseline BMI (kg/m^2^)= - 0.29 (95% CI – 0.40 to – 0.18), p = <0.001  Age of illness onset (years) = 0.04 (95% CI -0.33 to 0.41), p 0.85  Atypical class of antipsychotic (Y/N) = 1.29 (95% CI -0.66 to 3.24), p = 0.19  Anticholinergic drug prescribed (Y/N) = - 0.08 (95% CI -1.51 to 1.35), p = 0.91  Reduction in PANSS score total = 0.03 (95% CI 0.01 to 0.05), p = 0.002 |
| Pandit (2019) | Exploratory – range of candidate prognostic factors assessed | (1) Change in weight at 4 weeks  (2) Risk of clinically significant weight gain at 4 weeks | Adjusted - Multivariable linear + logistic regression | **Linear regression results (β) =**  Age (years) = - 0.07 (95% CI -0.13 to -0.01), p = 0.031  Gender (M/F) = 0.01 (95% CI -0.89 to 0.92), p = 0.982  Ethnicity (white vs. other) = 0.16 (95% CI -1.04 to 1.35), p = 0.798  Unemployed (Y/N)= 0.94 (95% CI 0.18 to 1.71), p = 0.016  Primary diagnosis (schizophreniform vs. schizophrenia) = 0.59 (95% CI -0.20 to 1.37)  Comorbid MDD (Y/N) = 1.61 (95% CI -3.10 to -0.12), p = 0.034  PANSS total score = -0.01 (95% CI -0.03 to 0.01), p = 0.380  Antipsychotic-naïve (Y/N) = -0.50 (95% CI -1.32 to 0.33), p = 0.380  Inpatient vs. outpatient care = -0.27 (95% CI -1.41 to 0.60), p = 0.537  Baseline body weight = -0.02 (95% CI -0.05 to 0.01), p=0.182  **Logistic regression results (OR) =**  Age (years) = 0.96 (95% CI 0.91 – 1.01), p = 0.106  Gender (M/F) = 0.94 (95% CI 0.46 to 1.89), p = 0.852  Ethnicity (white vs. other) = 1.34 (95% CI 0.52 to 3.44), p = 0.545  Unemployed (Y/N) = 2.83 (1.50 to 5.36), p = 0.001  Primary diagnosis (schizophreniform vs. schizophrenia) = 2.00 (95% CI 1.09 to 3.68), p = 0.025  Comorbid MDD = 0.39 (95% CI 0.08 to 1.79), p = 0.224  PANSS total score = 0.99 (95% CI 0.97 to 1.00), p = 0.085  Antipsychotic-naïve (Y/N) = 0.72 (95% CI 0.37 to 1.42), p = 0.345  Inpatient vs. outpatient care = 1.34 (95% CI 0.67 to 2.67), p = 0.407  Baseline body weight (kg) = 0.97 (95% CI 0.95 to 0.99), p = 0.032 |
| Perez-Iglesias (2014) | Exploratory – range of candidate prognostic factors assessed | Change in weight at 3,12 and 36 months | Adjusted - Multivariable linear regression | **Linear regression results (β) at 3 months =**  Olanzapine treatment assignment = 0.185 (95% CI 0.04 to 0.33), p = 0.014  Gender (M/F) = 0.288 (95% CI 0.13 to 0.44), p = <0.001  Baseline BMI (kg/m^2^) = -0.285 (95% CI -0.18 to -0.05), p = <0.001  **Linear regression results (β) at 12 months =**  Poor social functioning (deficit vs. no deficit) = 0.159 (95% CI 0.00 to 0.32), p = 0.052  **Linear regression results (β) at 36 months =**  Poor social functioning (deficit vs. no deficit) = 0.249 (95% CI 0.05 to 0.45) |
| Saddichha (2008) | Exploratory – range of candidate prognostic factors assessed | (1) Weight change at 6 weeks  (2) BMI change at 6 weeks  (3) Waist circumference change at 6 weeks | Unadjusted – (Pearson’s correlation analysis/ANOVA)  Adjusted – Multivariable linear regression | **Unadjusted analysis for change in BMI =**  Baseline weight, r = 0.248 (no standard error or confidence interval provided), p = <0.01  **Unadjusted analysis for change in waist circumference =**  Baseline waist circumference, r = -0.251 (no standard error or confidence interval provided), p<0.01  Baseline weight, r = -0.246 (no standard error or confidence interval provided), p<0.01  Gender (M/F) F test = 5.602 (no standard error or confidence interval provided), p<0.001  Linear regression results (β) for all outcomes only results of antipsychotic rx were described which were not under explicit investigation as part of study question due to established association. |
| Kang (2022) | Confirmatory – Olanzapine plasma concentration | (1) Change in weight at 8 weeks  (2) Change in BMI at 8 weeks | Unadjusted – Spearman’s rank correlation analysis  Adjusted – multivariable linear regression | **Unadjusted results for change in weight (rho) =**  Gender (M/F) = 0.086 (no standard error or confidence interval provided), p = >0.05  Smoking (Y/N) = 0.256 (no standard error or confidence interval provided), p = >0.05  Course of illness (years) = 0.021 (no standard error or confidence interval provided), p = >0.05  Olanzapine concentration (ng/ml) = 0.375 (no standard error or confidence interval provided), p = <0.01  Baseline BMI (kg/m^2^) = 0.987 (no standard error or confidence interval provided), p = <0.01  Fasting glucose (mmol/L) = 0.096 (no standard error or confidence interval provided), p = >0.05  Triglycerides (mmol/L) = -0.095 (no standard error or confidence interval provided), p = >0.05  Total cholesterol (mmol/L) = 0.114 (no standard error or confidence interval provided), p = >0.05  LDL-C (mmol/L) = 0.137 (no standard error or confidence interval provided), p = >0.05  HDL-C (mmol/L) = 0.377 (no standard error or confidence interval provided), p = <0.01  Baseline PANSS measurement = 0.126 (no standard error or confidence interval provided), p = >0.05  BMI rate change = 0.977(no standard error or confidence interval provided), p = <0.01  **Unadjusted results for change in BMI (rho) =**  Gender (M/F) = 0.148 (no standard error or confidence interval provided), p = >0.05  Smoking (Y/N = 0.275 (no standard error or confidence interval provided), p = >0.05  Course of illness (years) = 0.041 (no standard error or confidence interval provided), p = >0.05  Olanzapine concentration (ng/ml) = 0.365 (no standard error or confidence interval provided), p = <0.01  Baseline weight (kg) = 0.987 (no standard error or confidence interval provided), p = <0.01  Fasting glucose (mmol/L) = 0.087 (no standard error or confidence interval provided), p = >0.05  Triglycerides (mmol/L) = --0.137 (no standard error or confidence interval provided), p = >0.05  Total cholesterol (mmol/L) = 0.104 (no standard error or confidence interval provided), p = >0.05  LDL-C (mmol/L) = 0.147 (no standard error or confidence interval provided), p = >0.05  HDL-C (mmol/L) = 0.388 (no standard error or confidence interval provided), p = <0.01  Baseline PANSS measurement = 0.112 (no standard error or confidence interval provided), p = >0.05  BMI rate change = 0.988 (no standard error or confidence interval provided), p = <0.01  **Linear regression results (β) for change in weight =**  Olanzapine plasma concentration (ng/ml) = 0.376 (95% CI 0.08 to 0.67), p = 0.013  Smoking status (Y/N) = 0.130 (95% CI -0.15 to 0.41), p = 0.365  Course of illness (years) = 0.067 (95% CI -0.21 to 0.34), p = 0.631  Gender (M/F) = 0.065 (95% CI -0.2 to 0.33), p = 0.63  **Linear regression results (β) for change in BMI =**  Olanzapine plasma concentration (ng/ml) = 0.35 (95% CI 0.06 to 0.65), p = 0.019  Smoking status (Y/N) = 0.164 (95% CI -0.12 to 0.45), p = 0.254  Course of illness (years) = 0.096 (95% CI -0.18 to 0.37), p = 0.119  Gender (M/F) = 0.119 (95% CI -0.15 to 0.39), p = 0.387 |
| Rasmussen (2014) | Neocortical 5HT2A binding receptor profile | Change in weight over 24 weeks | Unadjusted – Spearman’s rank correlation analysis | **Unadjusted results for change in weight (rho) =**  5HT2A receptor binding capacity = 0.59 (no standard error or confidence interval provided), p =0.022 |
| Muntane (2022) | Exploratory – range of candidate prognostic factors assessed | (1) Change in BMI at 12 weeks  (2) Change in BMI at 52 weeks | Adjusted – multivariable linear regression | **Linear regression results (b) for change in BMI at 12 weeks =**  Age (years) = -0.04 (95% CI -0.12 to 0.04), p = 0.37  Gender (F/M) = -2.08 (95% CI 0.47 to 3.69), p = 0.01  Baseline BMI (kg/m^2^)= -0.60 (95% CI -0.80 to -0.40), p = <0.0001  Diagnosis (reference = schizophrenia diagnosis)  Delusional disorder = -0.61 (95% CI -10.29 to 9.07), p = 0.90  Schizoaffective disorder = -0.37 (95% CI -6.15 to 5.78), p = 0.91  Schizophreniform disorder = 0.91 (95% CI -0.81 to 2.63), p = 0.30  Brief psychotic disorder = -0.24 (95% CI -2.77 to 2.29), p = 0.85  Unspecified psychosis = 1.45 (95% CI -1.51 to 4.41), p = 0.34  Concomitant antidepressant use (Y/N) = 0.04 (95% CI -2.12 to 2.20), p = 0.97  **Linear regression (b) results for change in BMI at 52 weeks =**  Age (years) = -0.17 (95% CI -0.29 to -0.05), p = 0.0046  Gender (F/M) = 1.43 (95% CI -3.68 to 0.82), p =0.21  BMI increase at 3 months = 0.89 (95% CI 0.73 to 1.05), p = <0.001  Baseline BMI (kg/m^2^) = -0.13 (95% CI -0.40 to 0.14), p = 0.91 |
| Nielsen (2016) | Confirmatory - striatal reward activity   - Right-sided putamen - Left-sided putamen - Right ventral striatum - Left ventral striatum | Change in weight at 6 weeks | Adjusted – multivariable linear regression | **Linear regression results** **(β) for change in weight* =**  Left putamen reward activity = -0.69 (95% CI -1.73 to 0.34), p = 0.196  Right ventral striatal reward activity = -0.546 (95% CI -1.72 to 0.62), p = 0.367  Left ventral striatal reward activity = 0.128 (95% CI -1.14 to 1.40), p = 0.845  ***** Additional dataset received from authors |
| Homan (2019) | Confirmatory  (1) Striatal volume  (2) Striatal resting-state functional connectivity | Weight change over 12 weeks | Adjusted – multivariable linear regression | **Striatal volume**  **Linear regression results (β) for change in weight =**  Average putamen volume = 0.31 (95% CI 0.02 to 0.60), p = 0.036 |
| Liu (2022) | Exploratory – range of candidate prognostic factors assessed | Change in weight over 12 weeks | Adjusted – multivariable linear regression | **Linear regression results (β) for change in weight* =**  Age (years) = -0.07 (95% CI -0.09 to 0.04), p = 0.50  Education (years) = -0.22 (95% CI -0.36 to -0.02), p = 0.03  Smoking status (Y/N) = -0.04 (95% CI -2.85 to 1.88), p = 0.69  Baseline BMI (kg/m^2^) = -0.19 (95% CI -0.37 to 0.002), p = 0.05  Superoxide dismutase (U/ml) = -0.23 (95% -0.12 to -0.008), p = 0.025  Glutathione peroxidase (U/ml) = -0.24 (95% CI -0.37 to 0.02), p = 0.014  *results only reported for female participants in reporting. Not specified in statistical analysis plan. |
| Song (2014) | Pro-inflammatory cytokines   - IL-1B - IL-6 - TNF-alpha | Risk of clinically significant weight gain* over 24 weeks | Adjusted – multivariable logistic regression | **Logistic regression results (OR) for risk of clinically significant weight gain**  IL-1β = 0.91 (0.85-0.97), p = 0.005 |
| Yuan (2018) | Exploratory – range of candidate prognostic factors assessed | (1) Weight change over 24 weeks  (2) BMI Change over 24 weeks | Adjusted – multivariable linear regression | **Linear regression results for change in weight**  R^2^ for model including age, gender, smoking status and disease duration = 8.3%, F = 0.631, P = 0.677  **Linear regression results for change in BMI**  R^2^ for model including age, gender, smoking status and disease duration = 9%, F = 0.695, p = 0.631 |
| Lin (2021) | Confirmatory – Illness severity (positive symptoms) | BMI change over mean 6.04 years | Unadjusted – Pearson’s correlation analysis + univariable linear regression  Adjusted – multivariable linear regression | **Unadjusted results for change in BMI (r) =**  Positive symptom severity = -0.23, (no standard error or confidence interval provided), p = 0.30  Negative symptom severity = 0.25, (no standard error or confidence interval provided), p = 0.25  **Linear regression results (b) for change in BMI* =**  Positive symptom severity = 0.14 (95% CI 0.13 to 0.15), p <0.01  BMI at baseline (mg/k^2^) = 0.04 (95% CI 0.02 to 0.06), p <0.01  Age (years) = -0.004 (95% CI -0.01 to 0.01), p = 0.4237  Gender (M/F) = -0.04 (95% CI -0.16 to 0.08), p = 0.50  Duration of illness (years) = -0.004 (95% CI -0.01 to 0.01), p = 0.4237 |
| Medved (2009) | Exploratory – range of candidate prognostic factors assessed | (1) Change in waist circumference over 12 weeks  (2) Change in BMI over 12 weeks | Adjusted – Repeated measures ANOVA | No effects sizes reported for either outcome and not available from study author upon multiple requests. |
| Zhang (2003) | Exploratory – range of candidate prognostic factors assessed | BMI over 10 weeks | Adjusted – multivariable linear regression | No effects sizes reported and not available from study author upon multiple requests. |
| Verma (2009) | Exploratory – range of candidate prognostic factors assessed | Risk of clinically significant weight gain * over 24 weeks | Adjusted – multivariable logistic regression | **Logistic regression results for risk of clinically significant weight gain (OR)**  Age (years) = 0.6 (95% CI 0.6 to 0.9), p = 0.01  Gender (M/F) = 0.1 (95% CI 0 to 1.0), p = 0.05  Baseline BMI (kg/m^2^) = 0.8 (95% CI 0.6 to 1.0), p = 0.03  Cumulative antipsychotic exposure = 1.0 (95% CI 1.0-1.0), p = 0.36  % Change in PANSS positive score = 1.0 (95% CI 0.9 to 1.2), p = 0.77  Baseline low density lipoprotein cholesterol (LDL-C) (mmol/L) = 2.0 (95% CI 0.7 – 5.9), p = 0.22  Baseline triglycerides (mmol/L) = 4.1 (95% CI 1.0 to 15.2), p = 0.05  Baseline insulin (mmol/L) = 1.0 (95% CI 1.0 to 1.0), p = 0.87 |
| Arranz (2007) | Confirmatory - Antipsychotic formulation – standard vs. disintegrating | (1) Weight change over 6 weeks  (2) BMI change over 6 weeks | Analysis of Covariance (ANCOVA) | **Results for change in weight =**  Olanzapine standard tablets: mean difference = 6.4 (3.96)  Olanzapine disintegrating tablets: mean difference = 3.3 (2.93)  F = 7.7, p = 0.009  **Results for change in BMI =**  Olanzapine standard tablets: mean difference = 2.1 (1.06)  Olanzapine disintegrating tablets: mean difference = 1.1 (0.806)  F = 4.7, p = 0.036 |
| Li (2018) | Exploratory – range of candidate prognostic factors assessed | Change in weight over 12 weeks | Unadjusted – Spearman’s rank correlation analysis  Adjusted – multivariable linear regression | **Unadjusted results for change in weight (rho) =**  Gender (M/F) = 0.042 (no standard error or confidence interval provided), p = 0.471  Age (years) = -0.205 (no standard error or confidence interval provided), p = 0.00001  Duration of illness (years) = -0.188 (no standard error or confidence interval provided), p = 0.001  Baseline BMI (kg/m^2^) = -0.108 (no standard error or confidence interval provided), p = 0.065  Waist to hip ratio = -0.199 (no standard error or confidence interval provided), p = 0.008  Systolic blood pressure (mmHg) = -0.075 (no standard error or confidence interval provided), p = 0.206  Diastolic blood pressure (mmHg) = -0.124 (no standard error or confidence interval provided), p = 0.036  Triglycerides (mmol/L) = 0.010, (no standard error or confidence interval provided), p = 0.873  High-density lipoprotein cholesterol (mmol/L) = - 0.014 (no standard error or confidence interval provided), p = 0.808  Low-density lipoprotein cholesterol (mmol/L) = - 0.068 (no standard error or confidence interval provided), p = 0.248  Total cholesterol (mmol/L) = - 0.068 (no standard error or confidence interval provided), p = 0.460  Total plasma protein (g/l) = - 0.116 (no standard error or confidence interval provided), p = 0.050  Albumin (g/l) = -0.061 (no standard error or confidence interval provided),p = 0.304  Fasting plasma glucose (mmol/L) = - 0.144 (no standard error or confidence interval provided), p = 0.015  Urea (mmol/L) = -0.022 (no standard error or confidence interval provided), p = 0.714  C-reactive protein (CRP) (mg/L) = -0.087 (no standard error or confidence interval provided), p = 0.394  Thyroid Stimulating Hormone (TSH) (mIU/ml) = -0.138 (no standard error or confidence interval provided), p = 0.029  T3 (nmol/L) = - 0.120 (no standard error or confidence interval provided), p = 0.058  T4 (nmol/L) = - 0.154 (no standard error or confidence interval provided), p= 0.014  **Linear regression results (b) for change in weight =**  Age (years) = -0.02 (95% CI = -0.04 to 0.00), p = 0.107  Duration of illness (years) = 0.02 (95% CI = -0.01 to 0.05), p = 0.251  Waist to hip ratio = -2.45 (95% -4.83 to – 0.08), p = 0.044  Diastolic blood pressure (mmHg) = -0.002 (95% CI -0.03 to 0.02), p = 0.8146  Total plasma protein (g/l) = -0.004 (95% CI -0.04 to 0.03) ,p = 0.83  Fasting plasma glucose (mmol/L) = - 0.112 (95% CI -0.31 to 0.08), p = 0.26  Thyroid Stimulating Hormone (TSH) (mIU/ml) = -0.075 (95% CI -0.19 to 0.04), p = 0.192  T4 (nmol/L) = - 0.01 (95% CI -0.02 to 0.00), p = 0.038 |
| Chiliza (2015) | Exploratory – range of candidate prognostic factors assessed | Change in BMI at 52 weeks | Adjusted – multivariable linear regression | **Linear regression results (b) for change in BMI* =**  Age (years) = -0.002 (SE = 0.06), p = 0.97  Gender (M/F) = 0.89 (SE = 0.78), p = 0.25  Substance misuse history (Y/N) = - 2.249 (SE = 0.71), p = 0.002  Previous treatment (Y/N) = 0.62 (SE = 0.669), = 0.35  Baseline BMI (kg/m^2^) = -0.088 (SE = 0.09), p = 0.334  Baseline high-density cholesterol (mmol/L) = 0.157 (SE = 0.63), p = 0.80  Baseline low-density cholesterol (mmol/L) = 0.508 (SE = 0.67), p = 0.45  Baseline fasting glucose (mmol/L) = -0.44 (SE = 0.48), p = 0.35  Baseline prolactin (mIU/L) = 0.07 (SE = 0.04), p = 0.12  Baseline triglycerides (mmol/L) = -0.80 (SE = 0.711), p = 0.26  Baseline cholesterol (mmol/L) = -0.59, (SE = 0.60), p = 0.33  ***Full dataset retrieved from author** |
| Canal-Rivero (2020) | Exploratory – range of candidate prognostic factors assessed | Change in weight at 52 weeks | Adjusted – multivariable linear regression | **Linear regression results (b) for change in weight =**  Dietary habits at 3 months follow-up after FEP = 2.56 (95% CI 0.79 to 4.33), p = 0.0045  Baseline BMI (kg/m^2^) = -0.46 (95% CI -0.74 to - 0.17), p <0.01  Emerging adulthood (18-30 vs 30-60 years of age) = -2.76 (95% CI -5.34 to – 0.18), p = 0.04 |
| Huang (2020) | Confirmatory - early appetite increase | (1) Weight change over 12 weeks  (2) BMI change over 12 weeks | Adjusted - multivariable linear regression1 | **Linear regression results (β) for change in weight** = 0.67 (95% CI 0.31 to 1.03), p = 0.0003  **Linear regression results (β) for change in BMI)** = 0.63 (95% CI 0.28 to 0.98), p = 0.004  Prognostic factor = appetite increase (>10% increase in baseline appetite score vs. no) |
| Luckhoff (2020) | Confirmatory - Hippocampal subfield volume – anterior + posterior | Change in BMI at 52 weeks | Unadjusted – Spearman’s rank correlation analysis  Adjusted - multivariable linear regression | **Unadjusted estimates (r) =**  Anterior hippocampal volume = -0.10, p = 0.327  Posterior hippocampal volume = -0.07, p = 0.506  **Linear regression results (b) for change in BMI =**  Anterior hippocampal volume = 3.03 (95% CI = 0.11 - 5.95), p = 0.046  Posterior hippocampal volume = 2.18 (95% CI = -1.50 – 5.86), p = 0.250 |
| Zipursky (2005) | Exploratory – range of candidate prognostic factors assessed | Time to clinically significant weight gain assessed over 104 weeks | Adjusted - Cox regression | No numerical study results available in report or on request from study authors. |
| Garriga (2019) | Confirmatory – birth weight | Change in weight at 16 weeks | Adjusted - Linear mixed model analysis  Curvilinear regression analysis applied to assess for presence of quadratic relationship | **Linear regression results (β) for change in weight** = 0.219 (95% CI 0.04 to 0.40), p = 0.018*  **Curvilinear regression** **quadratic correlation =**  R^2^ = 0.127, β = 4.328, p = 0.002*  *Birth weight measured in kg |
| Lago (2021) | Confirmatory – cellular markers of metabolic syndrome on peripheral blood mononuclear cells (glucose transporter 1, insulin receptor, fatty acid translocase on CD4+ T cells, CD8+ T cells, CD4-8- T cells, B cells and monocytes) | Change in BMI at 12 weeks | Adjusted - multivariable linear regression | **Linear regression results (b) for change in BMI =**  CD36 expression on T cells CD4+ = 0.0148, SE = 0.005, p = 0.005  Insulin receptor expression on monocytes = 0.0038, SE = 0.00241, p = 0.119  CD36 expression on CD4- CD8- T cells = 0.00897, SE = 0.01, p = 0.419  Glucose transporter 1 expression on monocytes = 0.000385, SE = 0.00162, p = 0.814 |
| Zhou (2019) | Confirmatory - gender | BMI change over 12 weeks | Adjusted analysis – Generalized Additive Mixed Model (GAMM) | Results in graph form only. |
| Vázquez-Bourgon (2022) | Exploratory – range of candidate prognostic factors assessed | (1) Weight change at 52 weeks  (2) BMI change at 52 weeks | Adjusted analysis multivariable linear regression | Dataset provided reanalysed via preferred multivariable linear regression. Dataset found here - <http://doi.org/10.17632/b2h5gr9m3c.1> |
| β = standardized regression coefficient  b = unstandardized regression coefficient  ANOVA = Analysis of Variance  ANCOVA = Analysis of Covariance  OR = Odds Ratio  SE = Standard Error  *Clinically significant weight gain is defined as a 7 % or greater increase in baseline body weight. | | | | |

# Part 6 – Risk of bias assessment results


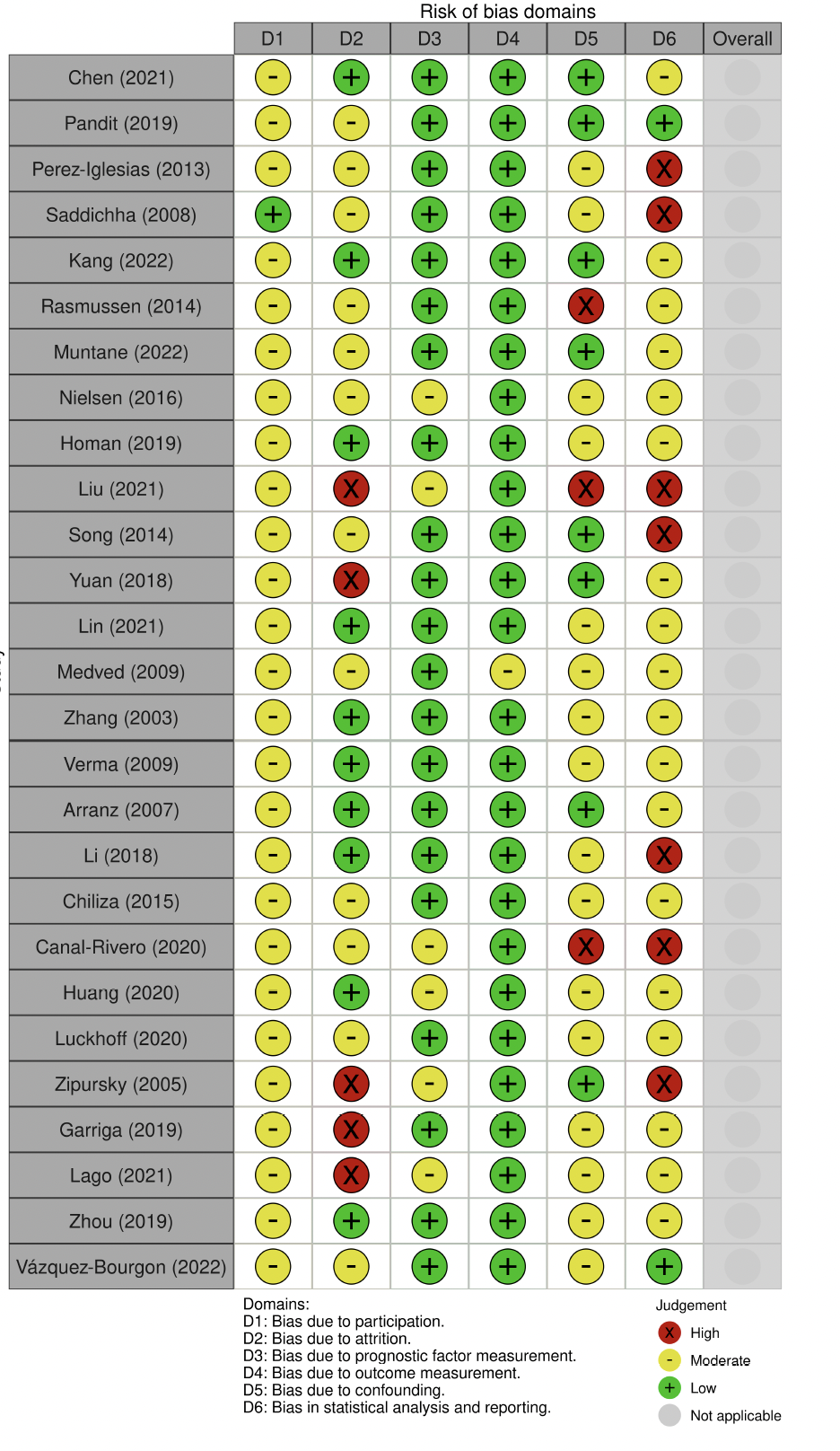


# Part 7 - GRADE prognostic factor-outcome assessments

| Quality level | Definition |
| --- | --- |
| Interpretations of GRADE quality ratings for each rating | |
| High | We are very confident that the true effect lies close to that of the estimate of the effect. |
| Moderate | We are moderately confident in the effect estimate: the true effect is likely to be close to the estimate of the effect, but there is a possibility that it is substantially different. |
| Low | Our confidence in the effect estimate is limited: the true may be substantially different from the estimate of the effect. |
| Very low | We have very little confidence in the effect estimate: the true effect is likely to be substantially different from the estimate of effect |

## Section 1 - GRADE tables for all prognostic factor-outcome associations assessed narratively only.

### Table 1 – Prognostic factors and association with risk clinically significant weight gain at all timepoints.

| **Outcome: Risk of clinically significant weight gain** | | | | | | | | | | | | | | | | |
| --- | --- | --- | --- | --- | --- | --- | --- | --- | --- | --- | --- | --- | --- | --- | --- | --- |
| Univariate Multivariate GRADE factors | | | | | | | | | | | | | | | | |
| Prognostic factor | Participant no. | Number of studies (+ study number 1-27) | + | 0 | - | + | 0 | - | Phase | Study limitations | Inconsistency | Indirectness | Imprecision | Publication bias | Reasons to upgrade? | Overall quality |
| Age (years) | 875 | 4 (2,4,16,23) | 0 1 0 | | | 1 2 0 | | | 1 | X | X | 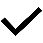 | 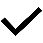 | 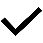 | No | Low |
| Gender (M/F) | 875 | 4 (2,4,16,23) | 0 1 0 | | | 1 2 0 | | | 1 | X | X | 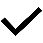 | X | 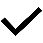 | No | Low |
| Ethnicity  (white vs. others) | 446 | 1 (2) | 0 0 0 | | | 0 1 0 | | | 1 | 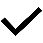 | Unclear | 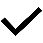 | 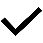 | Unclear | No | Low |
| Employment status (unemployed vs employed) | 446 | 1 (2) | 0 0 0 | | | 1 0 0 | | | 1 | 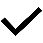 | Unclear | 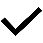 | 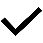 | Unclear | Yes | Moderate |
| Primary diagnosis | 819 | 3 (2,4,23) | 1 0 0 | | | 1 1 0 | | | 1 | X | X | 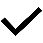 | X | 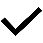 | No | Low |
| Comorbid Major Depressive Disorder (Y/N) | 446 | 1 (2) | 0 0 0 | | | 0 1 0 | | | 1 | 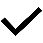 | Unclear | 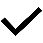 | X | Unclear | No | Low |
| Illness severity | 446 | 1 (2) | 0 0 0 | | | 0 1 0 | | | 1 | 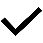 | Unclear | 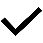 | 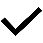 | Unclear | No | Low |
| Previous antipsychotic use (antipsychotic-naïve) | 709 | 2 (2,23) | 0 0 0 | | | 0 2 0 | | | 1 | X | 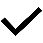 | X | X | Unclear | No | Very low |
| Inpatient vs outpatient care | 446 | 1 (2) | 0 0 0 | | | 0 1 0 | | | 1 | 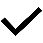 | Unclear | 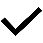 | X | Unclear | No | Low |
| Baseline bodyweight | 446 | 1 (2) | 0 0 0 | | | 0 0 1 | | | 1 | 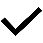 | Unclear | 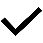 | 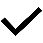 | Unclear | No | Low |
| Baseline BMI (kg/m^2^) | 166 | 2 (4,16) | 1 0 0 | | | 1 0 0 | | | 1 | X | 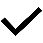 | 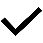 | X | X | No | Low |
| Pro-inflammatory cytokine - TNF-alpha | 78 | 1(11) | 0 1 0 | | | 0 1 0 | | | 2 | X | Unclear | X | Unclear | Unclear | No | Very low |
| Pro-inflammatory cytokine – ILB | 78 | 1(11) | 1 0 0 | | | 1 0 0 | | | 2 | X | Unclear | X | 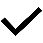 | Unclear | No | Very low |
| Pro-inflammatory cytokine - IL-6 | 78 | 1(11) | 1 0 0 | | | 0 1 0 | | | 2 | X | Unclear | X | Unclear | Unclear | No | Very low |
| Cumulative antipsychotic exposure | 56 | 1(16) | 0 0 0 | | | 0 1 0 | | | 1 | X | Not applicable | 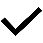 | X | Unclear | No | Very low |
| Response to antipsychotic treatment - positive symptoms | 56 | 1(16) | 0 0 0 | | | 0 1 0 | | | 1 | X | Not applicable | 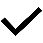 | 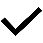 | Unclear | No | Low |
| Baseline low density lipoprotein  (LDL) cholesterol | 56 | 1(16) | 0 0 0 | | | 0 1 0 | | | 1 | X | Not applicable | 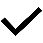 | X | Unclear | No | Very low |
| Baseline triglycerides | 56 | 1(16) | 0 0 0 | | | 1 0 0 | | | 1 | X | Not applicable | 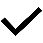 | X | Unclear | No | Very low |
| Baseline insulin secretion | 56 | 1(16) | 0 0 0 | | | 0 1 0 | | | 1 | X | Not applicable | 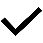 | 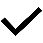 | Unclear | No | Low |
| Smoking status (Y/N) | 263 | 1 (23) | 0 0 0 | | | 0 1 0 | | | 1 | X | Not applicable | X | Unclear | Unclear | No | Very low |
| Premorbid functioning | 263 | 1 (23) | 0 0 0 | | | 0 1 0 | | | 1 | X | Not applicable | X | Unclear | Unclear | No | Very low |
| Age at illness onset | 263 | 1 (23) | 0 0 0 | | | 0 1 0 | | | 1 | X | Not applicable | X | Unclear | Unclear | No | Very low |
| Duration of illness | 263 | 1 (23) | 0 0 0 | | | 0 1 0 | | | 1 | X | Not applicable | X | Unclear | Unclear | No | Very low |

Phase = phase of investigation. For uni- and multivariate analyses: + number of events with a significant positive value, 0, number of non-significant events; - number of significant events with a negative value. For all other GRADE ratings:
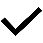
, no serious limitations; X, serious limitations; X, very serious limitations; unclear, unable to rate item based on available information. In the case of inconsistency, this was marked as ‘unclear’ in the case of single studies where the assessment between the PF and the outcome had not been replicated and study quality downgraded for this measure. This is based on guidance from Huguet et al who recommend downgrading the quality of evidence in such cases as this is an indicator that the literature in this area is not well established. Publication bias was also marked as unclear where the relationship between a prognostic factor and an outcome has only been assessed in a single study. Other parameters under assessment have been marked as unclear when data is missing from the study report and was not provided by study authors on request.

### Table 2 – Prognostic factors and association with change in waist circumference assessed at all timepoints.

| **Outcome: Change in waist circumference (cm)** | | | | | | | | | | | | | | | | |
| --- | --- | --- | --- | --- | --- | --- | --- | --- | --- | --- | --- | --- | --- | --- | --- | --- |
| Univariate Multivariate GRADE factors | | | | | | | | | | | | | | | | |
| Prognostic factor | Participant no. | Number of studies (+ study number 1-27) | + | 0 | - | + | 0 | - | Phase | Study limitations | Inconsistency | Indirectness | Imprecision | Publication bias | Reasons to upgrade? | Overall quality |
| Baseline waist circumference | 110 | 1 (4) | 0 0 1 | | | 0 0 0 | | | 1 | X | Unclear | 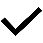 | Unclear | Unclear | No | Very low |
| Baseline weight | 110 | 1 (4) | 0 0 1 | | | 0 0 0 | | | 1 | X | Unclear | 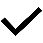 | Unclear | Unclear | No | Very low |
| Baseline BMI (kg/m^2^) | 110 | 1 (4) | 0 1 0 | | | 0 0 0 | | | 1 | X | Unclear | 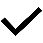 | Unclear | Unclear | No | Very low |
| Gender (M/F) | 110 | 1 (4) | 1 0 0 | | | 0 0 0 | | | 1 | X | Unclear | 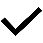 | Unclear | Unclear | No | Very low |
| Family history of diabetes mellitus (Y/N) | 94 | 1 (14) | 0 0 0 | | | 1 0 0 | | | 1 | X | Unclear | X | Unclear | Unclear | No | Low |
| Age* | 94 | 1 (14) | Unclear | | | Unclear | | | 1 | X | Unclear | X | Unclear | Unclear | No | Very low |
| Family history of cerebrovascular disease* (Y/N) | 94 | 1 (14) | Unclear | | | Unclear | | | 1 | X | Unclear | X | Unclear | Unclear | No | Very low |
| Family history of obesity* (Y/N) | 94 | 1 (14) | Unclear | | | Unclear | | | 1 | X | Unclear | X | Unclear | Unclear | No | Very low |
| Illness severity* | 94 | 1 (14) | Unclear | | | Unclear | | | 1 | X | Unclear | X | Unclear | Unclear | No | Very low |
| Smoking history* (Y/N) | 94 | 1 (14) | Unclear | | | Unclear | | | 1 | X | Unclear | X | Unclear | Unclear | No | Very low |
| Diagnosis* | 94 | 1 (14) | Unclear | | | Unclear | | | 1 | X | Unclear | X | Unclear | Unclear | No | Very low |
| Duration of illness* | 94 | 1 (14) | Unclear | | | Unclear | | | 1 | X | Unclear | X | Unclear | Unclear | No | Very low |

Phase = phase of investigation. For uni- and multivariate analyses: + number of events with a significant positive value, 0, number of non-significant events; - number of significant events with a negative value. For all other GRADE ratings:
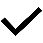
, no serious limitations; X, serious limitations; X, very serious limitations; unclear, unable to rate item based on available information. In the case of inconsistency, this was marked as ‘unclear’ in the case of single studies where the assessment between the PF and the outcome had not been replicated and study quality downgraded for this measure. This is based on guidance from Huguet et al who recommend downgrading the quality of evidence in such cases as this is an indicator that the literature in this area is not well established. Publication bias was also marked as unclear where the relationship between a prognostic factor and an outcome has only been assessed in a single study. Other parameters under assessment have been marked as unclear when data is missing from the study report and was not provided by study authors on request.

*Numerical results not reported or provided by study author on request

Y/N = Yes/No

### Table 3 – Prognostic factors and association with change in weight at all timepoints.

| **Outcome: Change in weight** | | | | | | | | | | | | | | | | |
| --- | --- | --- | --- | --- | --- | --- | --- | --- | --- | --- | --- | --- | --- | --- | --- | --- |
| Univariate Multivariate GRADE Factors | | | | | | | | | | | | | | | | |
| Prognostic factor | Participant number | Number of studies (+ study number 1-27) | + | 0 | - | + | 0 | - | Phase | Study limitations | Inconsistency | Indirectness | Imprecision | Publication bias* | Reasons to rate up? | Overall quality |
| Age | 601 | 3 (8,10,27) | 0 0 0 | | | 1 0 2 | | | 1 | X | X | 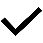 | 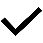 | 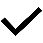 | No | Low |
| Diagnosis (schizophreniform vs. schizophrenia) | 446 | 1 (2) | 0 0 0 | | | 0 1 0 | | | 1 | 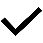 | Unclear | 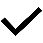 | 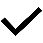 | Unclear | No | Low |
| Education | 751 | 2 (1,10) | 1 0 0 | | | 0 1 1 | | | 1 | X | 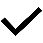 | 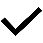 | 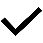 | Unclear | No | Low |
| Age at illness onset | 526 | 1 (1) | 0 1 0 | | | 0 1 0 | | | 1 | 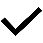 | Unclear | 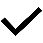 | 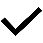 | Unclear | No | Low |
| Antipsychotic prescription | 69 | 1(8) | 0 0 0 | | | 0 0 1 | | | 1 | X | Unclear | 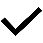 | 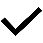 | Unclear | No | Low |
| Duration of illness | 526 | 1 (1) | 1 0 0 | | | 0 0 0 | | | 1 | X | Unclear | 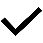 | 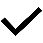 | Unclear | No | Very low |
| Anticholinergic co-prescription | 526 | 1 (1) | 0 0 0 | | | 0 1 0 | | | 1 | X | Unclear | 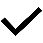 | 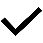 | Unclear | No | Low |
| Baseline hip circumference | 526 | 1 (1) | 0 0 1 | | | 0 0 0 | | | 1 | X | Unclear | 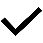 | Unclear | Unclear | No | Low |
| Duration of untreated psychosis | 225 | 2 (3,5) | 1 0 0 | | | 0 2 0 | | | 1 | X | 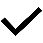 | 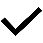 | 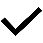 | X | No | Very low |
| Social functioning | 174 | 1 (3) | 0 0 0 | | | 1 0 0 | | | 1 | X | Unclear | 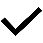 | 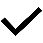 | Unclear | No | Very low |
| Response to antipsychotic treatment - positive symptoms | 700 | 2 (1,3) | 1 0 0 | | | 1 0 0 | | | 1 + 2 | X | 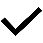 | 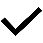 | 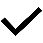 | Assumed as no evidence of not – as per 2013 advised approach | No | Very low |
| Response to antipsychotic treatment - negative symptoms | 700 | 2 (1,3) | 1 0 0 | | | 0 0 1 | | | 1 + 2 | X | 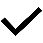 | 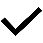 | X | Unclear | No | Very low |
| Response to antipsychotic treatment - general psychopathology | 526 | 1 (1) | 1 0 0 | | | 0 0 0 | | | 2 | 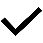 | Unclear | 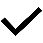 | 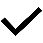 | Unclear | No | Low |
| Response to antipsychotic treatment - total psychopathology | 526 | 1 (1) | 1 0 0 | | | 1 0 0 | | | 2 | 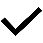 | Unclear | 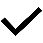 | 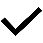 | Unclear | No | Moderate |
| Socioeconomic status | 174 | 1(1) | 0 0 0 | | | 0 1 0 | | | 1 | X | Unclear | 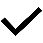 | Unclear | Unclear | No | Very low |
| Antipsychotic plasma concentration | 51 | 1 (5) | 1 0 0 | | | 1 0 0 | | | 2 | X | Unclear | 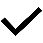 | 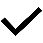 | Unclear | No | Moderate |
| BMI rate change | 51 | 1 (5) | 1 0 0 | | | 0 0 0 | | | 1 | X | Unclear | 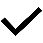 | X | Unclear | Yes | Low |
| Illness severity | 566 | 3 (2,5,8) | 0 2 0 | | | 0 1 0 | | | 1 | X | 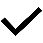 | 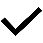 | 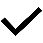 | X | No | Low |
| Baseline fasting glucose | 51 | 1 (5) | 0 1 0 | | | 0 0 0 | | | 1 | X | Unclear | 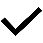 | X | Unclear | No | Very low |
| Baseline triglycerides | 51 | 1 (5) | 0 1 0 | | | 0 0 0 | | | 1 | X | Unclear | 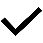 | X | Unclear | No | Very low |
| Baseline total cholesterol | 51 | 1 (5) | 0 1 0 | | | 0 0 0 | | | 1 | X | Unclear | 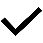 | X | Unclear | No | Very low |
| Baseline low density lipoprotein (LDL) cholesterol | 51 | 1 (5) | 0 1 0 | | | 0 0 0 | | | 1 | X | Unclear | 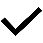 | X | Unclear | No | Very low |
| Baseline high density lipoprotein (HDL) cholesterol | 51 | 1 (5) | 1 0 0 | | | 0 0 0 | | | 1 | X | Unclear | 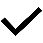 | X | Unclear | No | Very low |
| 5HT-2A receptor binding profile | 30 | 1 (6) | 1 0 0 | | | 0 0 0 | | | 2 | X | Unclear | X | X | Unclear | No | Very low |
| Striatal reward activity | 69 | 1(8) | 0 0 0 | | | 1 0 0 | | | 2 | X | Unclear | 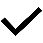 | X | Unclear | No | Low |
| Striatal functional connectivity | 81 | 1(9) | 0 0 0 | | | 1 0 0 | | | 2 | X | Unclear | X | 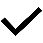 | Unclear | No | Low |
| Striatal volume | 81 | 1(9) | 0 0 0 | | | 1 0 0 | | | 2 | X | Unclear | X | 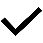 | Unclear | No | Low |
| Superoxide dismutase (SOD) | 293 | 1 (10) | 0 0 0 | | | 0 0 1 | | | 2 | X | Unclear | X | X | Unclear | No | Very low |
| Catalase (CAT) | 293 | 1 (10) | 0 0 0 | | | Results not reported | | | 2 | X | Unclear | X | Unclear | Unclear | No | Very low |
| Glutathione peroxidase (GPx) | 293 | 1 (10) | 0 0 0 | | | 0 0 1 | | | 2 | X | Unclear | X | X | Unclear | No | Very low |
| Malondialdehyde (MDA) | 293 | 1 (10) | 0 0 0 | | | Results not reported | | | 2 | X | Unclear | X | Unclear | Unclear | No | Very low |
| Antipsychotic formulation – standard vs. disintegrating tablets | 38 | 1 (17) | 0 0 0 | | | 1 0 0 | | | 2 | 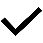 | Unclear | 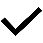 | X | Unclear | No | Low |
| Dietary habits at 3 months | 596 | 1 (20) | 0 0 0 | | | 1 0 0 | | | 1 | X | Unclear | 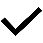 | 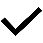 | Unclear | No | Very low |
| Early appetite increase | 33 | 1(21) | 1 0 0 | | | 1 0 0 | | | 2 | X | Unclear | 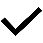 | 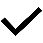 | Unclear | No | Low |
| Birth weight | 23 | 1 (24) | 1 0 0 | | | 1 0 0 | | | 2 | X | Unclear | X | 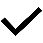 | Unclear | No | Very low |
| Antipsychotic dose | 23 | 1 (24) | 0 1 0 | | | 0 0 0 | | | 1 | X | Unclear | X | Unclear | Unclear | No | Very low |
| Positive symptom burden | 69 | 2 (8) | 0 1 0 | | | 0 0 0 | | | 1 | X | Unclear | 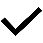 |  | Unclear | No | Low |
| Negative symptom burden | 69 | 2 (8) | 0 1 0 | | | 0 0 0 | | | 1 | X | Unclear |  |  | Unclear | No | Low |
| Employment status | 446 | 1(2) | 0 0 0 | | | 1 0 0 | | | 1 |  | Unclear |  |  | Unclear | Yes | Moderate |
| Comorbid Major Depressive Disorder | 446 | 1(2) | 0 0 0 | | | 1 0 0 | | | 1 |  | Unclear |  | X | Unclear | No | Low |
| Previous antipsychotic use (antipsychotic-naïve) | 446 | 1(2) | 0 0 0 | | | 0 1 0 | | | 1 |  | Unclear |  |  | Unclear | No | Moderate |
| Inpatient vs. outpatient care | 446 | 1(2) | 0 0 0 | | | 0 1 0 | | | 1 |  | Unclear |  |  | Unclear | No | Low |

Phase = phase of investigation. For uni- and multivariate analyses: + number of events with a significant positive value, 0, number of non-significant events; - number of significant events with a negative value. For all other GRADE ratings: , no serious limitations; X, serious limitations; X, very serious limitations; unclear, unable to rate item based on available information. In the case of inconsistency, this was marked as ‘unclear’ in the case of single studies where the assessment between the PF and the outcome had not been replicated and study quality downgraded for this measure. This is based on guidance from Huguet et al who recommend downgrading the quality of evidence in such cases as this is an indicator that the literature in this area is not well established. Publication bias was also marked as unclear where the relationship between a prognostic factor and an outcome has only been assessed in a single study. Other parameters under assessment have been marked as unclear when data is missing from the study report and was not provided by study authors on request.

### Table 4 – Prognostic factors and association with change in BMI at all timepoints.

| **Outcome:** Change in BMI | | | | | | | | | | | | | | | | |
| --- | --- | --- | --- | --- | --- | --- | --- | --- | --- | --- | --- | --- | --- | --- | --- | --- |
| Univariate Multivariate GRADE factors | | | | | | | | | | | | | | | | |
| Prognostic factor | Participant no. | Number of studies (+ study number 1-27) | + | 0 | - | + | 0 | - | Phase | Study limitations | Inconsistency | Indirectness | Imprecision | Publication bias | Reasons to upgrade? | Overall quality |
| Baseline waist circumference | 110 | 1(4) | 0 1 0 | | | 0 0 0 | | | 1 | X | Unclear |  | Unclear | Unclear | No | Very low |
| Baseline weight | 161 | 2 (4,5) | 2 0 0 | | | 0 0 0 | | | 1 |  |  |  |  | X | No | Low |
| Antipsychotic plasma concentration | 51 | 1 (5) | 1 0 0 | | | 1 0 0 | | | 2 | X | Unclear |  |  | Unclear | No | Moderate |
| Smoking status (Y/N) | 131 | 2 (5,12) | 0 2 0 | | | 0 1 0 | | | 1 | X |  | X |  | X | No | Low |
| Duration of untreated psychosis | 73 | 3 (5,13) | 1 0 0 | | | 1 1 0 | | | 1 | X | X | X |  | X | No | Low |
| Illness severity | 168 | 2 (5,15) | 0 1 0 | | | 0 2 0 | | | 1 | X |  |  |  | Unclear | No | Low |
| BMI rate change | 51 | 1 (5) | 1 0 0 | | | 0 0 0 | | | 1 |  | Unclear |  |  | Unclear | Yes | Low |
| Baseline fasting glucose | 480 | 3 (5,18,19) | 1 1 0 | | | 0 2 0 | | | 1 | X |  | X |  | X | No | Low |
| Baseline triglycerides | 480 | 3 (5,18,19) | 0 2 0 | | | 0 1 0 | | | 1 | X |  | X |  | X | No | Very low |
| Baseline total cholesterol | 480 | 3 (5,18,19) | 0 2 0 | | | 0 1 0 | | | 1 | X |  | X |  | X | No | Very low |
| Baseline low density lipoprotein (LDL) cholesterol | 480 | 3 (5,18,19) | 0 2 0 | | | 0 1 0 | | | 1 | X |  | X |  | X | No | Very low |
| Baseline high density lipoprotein (HDL) cholesterol | 480 | 3 (5,18,19) | 1 1 0 | | | 0 1 0 | | | 1 | X | X | X |  | X | No | Very low |
| Neocortical 5HT-2A receptor binding capacity (pre-antipsychotic) | 30 | 1 (6) | 1 0 0 | | | 0 0 0 | | | 2 | X | Unclear | X | Unclear | Unclear | No | Very low |
| Age (years) | 307 | 1 (27) | 0 0 0 | | | 1 0 0 | | | 1 | X | Unclear |  |  | Unclear | No | Low |
| Diagnosis | 381 | 1 (7) | 0 0 0 | | | 0 1 0 | | | 1 | X | Unclear |  | X | Unclear | No | Low |
| Antipsychotic dose | 498 | 2 (7,15) | 0 0 0 | | | 0 2 0 | | | 1 | X |  |  | Unclear | Unclear | No | Low |
| Concomitant antidepressant use | 381 | 1 (7) | 0 0 0 | | | 0 1 0 | | | 1 | X | Unclear |  |  | Unclear | No | Low |
| Trend of early BMI increase | 432 | 2 (5,7) | 1 0 0 | | | 1 0 0 | | | 1 | X |  |  |  | Unclear | Yes | Moderate |
| Duration of illness | 515 | 4 (12,13,15,18) | 0 0 1 | | | 0 4 0 | | | 1 | X |  | X |  | X | No | Low |
| Illness severity - positive symptoms | 22 | 1 (13) | 1 0 0 | | | 1 0 0 | | | 2 | X | Unclear |  |  | Unclear | No | Low |
| Illness severity - negative symptoms | 22 | 1 (13) | 1 0 0 | | | 0 0 0 | | | 2 | X | Unclear |  | Unclear | Unclear | No | Very low |
| Family history of diabetes | 94 | 1 (14) | 0 0 0 | | | 1 0 0 | | | 1 | X | Unclear | X | Unclear | Unclear | No | Low |
| Response to antipsychotic treatment - total psychopathology | 117 | 1(15) | 0 0 0 | | | 0 1 0 | | | 1 | X | Unclear |  | x | Unclear | No | Very low |
| Antipsychotic formulation | 38 | 1 (17) | 0 0 0 | | | 1 0 0 | | | 2 |  | Unclear |  | X | Unclear | No | Low |
| Waist-hip ratio | 296 | 1 (18) | 0 0 1 | | | 0 0 1 | | | 1 | X | Unclear | X | X | Unclear | No | Very low |
| Diastolic blood pressure | 296 | 1 (18) | 1 0 0 | | | 0 1 0 | | | 1 | X | Unclear | X |  | Unclear | No | Very low |
| Total protein | 296 | 1 (18) | 1 0 0 | | | 0 1 0 | | | 1 | X | Unclear | X |  | Unclear | No | Very low |
| Thyroid Stimulating Hormone (TSH) | 296 | 1 (18) | 1 0 0 | | | 0 1 0 | | | 1 | X | Unclear | X |  | Unclear | No | Very low |
| Thyroxine (T4) | 296 | 1 (18) | 1 0 0 | | | 1 0 0 | | | 1 | X | Unclear | X |  | Unclear | No | Very low |
| Albumin | 296 | 1 (18) | 0 1 0 | | | 0 0 0 | | | 1 | X | Unclear | X |  | Unclear | No | Very low |
| Systolic blood pressure | 296 | 1 (18) | 0 1 0 | | | 0 0 0 | | | 1 | X | Unclear | X |  | Unclear | No | Very low |
| C-Reactive Protein (CRP) | 296 | 1 (18) | 0 1 0 | | | 0 0 0 | | | 1 | X | Unclear | X |  | Unclear | No | Very low |
| T3 (Triiodothyronine) | 296 | 1 (18) | 0 1 0 | | | 0 0 0 | | | 1 | X | Unclear | X |  | Unclear | No | Very low |
| Urea | 296 | 1 (18) | 0 1 0 | | | 0 0 0 | | | 1 | X | Unclear | X |  | Unclear | No | Very low |
| Prolactin | 133 | 1 (19) | 0 0 0 | | | 0 1 0 | | | 1 | X | Unclear | X |  | Unclear | No | Low |
| Previous antipsychotic treatment | 223 | 2(19,22) | 0 0 0 | | | 1 1 0 | | | 1 | X | X | X | X | X | No | Very low |
| Substance abuse history | 133 | 1 (19) | 0 0 0 | | | 1 0 0 | | | 1 | X | Unclear | X | X | Unclear | No | Low |
| Early appetite increase | 33 | 1(21) | 1 0 0 | | | 1 0 0 | | | 1 | X | Unclear |  |  | Unclear | No | Low |
| Hippocampal subfield volume | 90 | 1(22) | 0 2 0 | | | 1 1 0 | | | 2 | X | Unclear | X | X | X | No | Low |
| Substance misuse (active) | 90 | 1 (22) | 0 0 0 | | | 1 0 0 | | | 1 | X | Unclear | X | X | X | No | Very low |
| Peripheral blood mononuclear cell subtypes | 58 | 1 (25) | 0 0 0 | | | 3 0 1 | | | 2 | X | Unclear | X |  | Unclear | No | Low |

Phase = phase of investigation. For uni- and multivariate analyses: + number of events with a significant positive value, 0, number of non-significant events; - number of significant events with a negative value. For all other GRADE ratings: , no serious limitations; X, serious limitations; X, very serious limitations; unclear, unable to rate item based on available information. In the case of inconsistency, this was marked as ‘unclear’ in the case of single studies where the assessment between the PF and the outcome had not been replicated and study quality downgraded for this measure. This is based on guidance from Huguet et al who recommend downgrading the quality of evidence in such cases as this is an indicator that the literature in this area is not well established. Publication bias was also marked as unclear where the relationship between a prognostic factor and an outcome has only been assessed in a single study. Other parameters under assessment have been marked as unclear when data is missing from the study report and was not provided by study authors on request.

## Section 2 - GRADE tables for all prognostic factor-outcome associations assessed via adjusted meta-analysis.

### Table 1 – Prognostic factors and association with change in BMI assessed at 52 weeks+

| **Outcome:** Change in BMI (adjusted) at 52 weeks + | | | | | | | | | | | | |
| --- | --- | --- | --- | --- | --- | --- | --- | --- | --- | --- | --- | --- |
| GRADE Factors | | | | | | | | | | | | |
| Prognostic factor | Number of participants | Number of studies (+ study number 1-27) | Number of cohorts | Estimated effect size (95% confidence interval) | Phase | Study limitations | Inconsistency | Indirectness | Imprecision | Publication bias | Reasons to rate up? | Overall quality |
| Gender (M/F) | 536 | 3 (7,13,19) | 3 | -0.04 (-0.347 – 0.270) | 1 | X |  |  | X |  | No | Low |
| Age  (years) | 626 | 4 (7,13,19,22) | 4 | -0.04 (-0.157-0.07) | 1 | X |  |  |  |  | No | Moderate |
| Baseline BMI (kg/m^2^) | 536 | 3 (7,13,19) | 3 | -0.01 (-0.225 – 0.2) | 1 | X |  |  | X | X | No | Low |

Phase = phase of investigation. For uni- and multivariate analyses: + number of events with a significant positive value, 0, number of non-significant events; - number of significant events with a negative value. For all other GRADE ratings: , no serious limitations; X, serious limitations; X, very serious limitations; unclear, unable to rate item based on available information. In the case of inconsistency, this was marked as ‘unclear’ in the case of single studies where the assessment between the PF and the outcome had not been replicated and study quality downgraded for this measure. This is based on guidance from Huguet et al who recommend downgrading the quality of evidence in such cases as this is an indicator that the literature in this area is not well established. Publication bias was also marked as unclear where the relationship between a prognostic factor and an outcome has only been assessed in a single study. Other parameters under assessment have been marked as unclear when data is missing from the study report and was not provided by study authors on request.

### Table 2 – Prognostic factors and association with change in weight assessed at 0-12 weeks.

###

| **Outcome: Change in weight at 12 weeks** | | | | | | | | | | | | |
| --- | --- | --- | --- | --- | --- | --- | --- | --- | --- | --- | --- | --- |
| GRADE Factors | | | | | | | | | | | | |
| Prognostic factor | Number of participants | Number of studies (+ study number 1-27) | Number of cohorts | Estimated effect size (95% confidence interval) | Phase | Study limitations | Inconsistency | Indirectness | Imprecision | Publication bias | Reasons to rate up? | Overall quality |
| Gender (M/F) | 1197 | 4 (1,2,3,5) | 4 | 0.236 (-0.086 to 0.0558) | 1 | X |  |  |  |  | No | Low |
| Age  (years) | 1041 | 3 (1,2,8) | 3 | -0.061 (-0.119 to -0.003) | 1 | X |  |  |  | X | No | Moderate |

Phase = phase of investigation. For uni- and multivariate analyses: + number of events with a significant positive value, 0, number of non-significant events; - number of significant events with a negative value. For all other GRADE ratings: , no serious limitations; X, serious limitations; X, very serious limitations; unclear, unable to rate item based on available information. In the case of inconsistency, this was marked as ‘unclear’ in the case of single studies where the assessment between the PF and the outcome had not been replicated and study quality downgraded for this measure. This is based on guidance from Huguet et al who recommend downgrading the quality of evidence in such cases as this is an indicator that the literature in this area is not well established. Publication bias was also marked as unclear where the relationship between a prognostic factor and an outcome has only been assessed in a single study. Other parameters under assessment have been marked as unclear when data is missing from the study report and was not provided by study authors on request.

***References***

1. Chen YQ, Li XR, Zhang L, Zhu WB, Wu YQ, Guan XN, et al. Therapeutic Response Is Associated With Antipsychotic-Induced Weight Gain in Drug-Naive First-Episode Patients With Schizophrenia: An 8-Week Prospective Study. J Clin Psychiatry. 2021 May 11;82(3).
2. Pandit R, Cianci D, Ter Hark SE, Winter-van Rossum I, Ebdrup BH, Broberg BV, et al. Phenotypic factors associated with amisulpride-induced weight gain in first-episode psychosis patients (from the OPTiMiSE cohort). Acta Psychiatr Scand. 2019 Sep;140(3):283–90.
3. Perez-Iglesias R, Crespo-Facorro B, Martinez-Garcia O, Ramirez-Bonilla ML, Alvarez-Jimenez M, Pelayo-Teran JM, et al. Weight gain induced by haloperidol, risperidone and olanzapine after 1 year: findings of a randomized clinical trial in a drug-naïve population. Schizophr Res. 2008 Feb;99(1–3):13–22.
4. Saddichha S, Ameen S, Akhtar S. Predictors of antipsychotic-induced weight gain in first-episode psychosis: conclusions from a randomized, double-blind, controlled prospective study of olanzapine, risperidone, and haloperidol. J Clin Psychopharmacol. 2008 Feb;28(1):27–31.
5. Kang D, Lu J, Liu W, Shao P, Wu R. Association between olanzapine concentration and metabolic dysfunction in drug-naive and chronic patients: similarities and differences. Schizophrenia [Internet]. 2022 Feb 28 [cited 2022 Dec 11];8(1):9. Available from: https://www.ncbi.nlm.nih.gov/pmc/articles/PMC8885747/
6. Rasmussen H, Ebdrup BH, Oranje B, Pinborg LH, Knudsen GM, Glenthøj B. Neocortical serotonin2A receptor binding predicts quetiapine associated weight gain in antipsychotic-naive first-episode schizophrenia patients. Int J Neuropsychopharmacol. 2014 Nov;17(11):1729–36.
7. Muntané G, Vázquez-Bourgon J, Sada E, Martorell L, Papiol S, Bosch E, et al. Leveraging genetics to improve Body Mass Index increase prediction in the first-episode of psychosi [Internet]. 2022 [cited 2022 Dec 7]. Available from: https://doi.org/10.1101/2022.02.15.22270982
8. Nielsen MØ, Rostrup E, Wulff S, Glenthøj B, Ebdrup BH. Striatal Reward Activity and Antipsychotic-Associated Weight Change in Patients With Schizophrenia Undergoing Initial Treatment. JAMA Psychiatry. 2016 Feb;73(2):121–8.
9. Homan P, Argyelan M, Fales CL, Barber AD, DeRosse P, Szeszko PR, et al. Striatal volume and functional connectivity correlate with weight gain in early-phase psychosis. Neuropsychopharmacol Off Publ Am Coll Neuropsychopharmacol. 2019 Oct;44(11):1948–54.
10. Liu H, Yu R, Gao Y, Li X, Guan X, Thomas K, et al. Antioxidant enzymes and weight gain in drug-naive first episode schizophrenia patients treated with risperidone for 12 weeks: a prospective longitudinal study. Curr Neuropharmacol. 2021 Sep 19;
11. Song X, Fan X, Li X, Zhang W, Gao J, Zhao J, et al. Changes in pro-inflammatory cytokines and body weight during 6-month risperidone treatment in drug naïve, first-episode schizophrenia. Psychopharmacology (Berl). 2014 Jan;231(2):319–25.
12. Yuan X, Zhang P, Wang Y, Liu Y, Li X, Kumar BU, et al. Changes in metabolism and microbiota after 24-week risperidone treatment in drug naïve, normal weight patients with first episode schizophrenia. Schizophr Res. 2018 Nov;201:299–306.
13. Lin SH, Tseng HH, Tsai HC, Chi MH, Lee IH, Chen PS, et al. Positive Symptoms in Antipsychotic-naïve Schizophrenia are Associated with Increased Body Mass Index after Treatment. Clin Psychopharmacol Neurosci Off Sci J Korean Coll Neuropsychopharmacol. 2021 Feb 28;19(1):155–9.
14. Medved V, Kuzman M, Jovanovic N, Grubisin J, Kuzman T. Metabolic syndrome in female patients with schizophrenia treated with second generation antipsychotics: a 3-month follow-up. J Psychopharmacol (Oxf) [Internet]. 2009 Nov 1 [cited 2023 Jan 4];23(8):915–22. Available from: <https://doi.org/10.1177/0269881108093927>
15. Zhang JP, Lencz T, Zhang RX, Nitta M, Maayan L, John M, et al. Pharmacogenetic Associations of Antipsychotic Drug-Related Weight Gain: A Systematic Review and Meta-analysis. Schizophr Bull. 2016 Nov;42(6):1418–37.
16. Verma S, Liew A, Subramaniam M, Poon LY. Effect of treatment on weight gain and metabolic abnormalities in patients with first-episode psychosis. Aust N Z J Psychiatry. 2009 Sep;43(9):812–7.
17. Arranz B, San L, Dueñas RM, Centeno M, Ramirez N, Salavert J, et al. Lower weight gain with the orally disintegrating olanzapine than with standard tablets in first-episode never treated psychotic patients. Hum Psychopharmacol. 2007 Jan;22(1):11–5.
18. Li S, Gao Y, Lv H, Zhang M, Wang L, Jiang R, et al. T4 and waist:hip ratio as biomarkers of antipsychotic-induced weight gain in Han Chinese inpatients with schizophrenia. Psychoneuroendocrinology. 2018 Feb;88:54–60.
19. Chiliza B, Asmal L, Oosthuizen P, van Niekerk E, Erasmus R, Kidd M, et al. Changes in body mass and metabolic profiles in patients with first-episode schizophrenia treated for 12 months with a first-generation antipsychotic. Eur Psychiatry J Assoc Eur Psychiatr. 2015 Feb;30(2):277–83.
20. Canal-Rivero M, Ruiz-Veguilla M, Labad J, Ayesa-Arriola R, Vázquez-Bourgon J, Mayoral-van Son J, et al. Predictors of weight acquisition induced by antipsychotic treatment and its relationship with age in a sample of first episode non-affective psychosis patients: A three-year follow-up study. Schizophr Res. 2020 Aug;222:462–4.
21. Huang J, Hei GR, Yang Y, Liu CC, Xiao JM, Long YJ, et al. Increased Appetite Plays a Key Role in Olanzapine-Induced Weight Gain in First-Episode Schizophrenia Patients. Front Pharmacol. 2020;11:739.
22. Luckhoff HK, du Plessis S, Kilian S, Asmal L, Scheffler F, Phahladira L, et al. Hippocampal subfield volumes and change in body mass over 12 months of treatment in first-episode schizophrenia spectrum disorders. Psychiatry Res Neuroimaging. 2020 Jun 30;300:111084.
23. Zipursky RB, Gu H, Green AI, Perkins DO, Tohen MF, McEvoy JP, et al. Course and predictors of weight gain in people with first-episode psychosis treated with olanzapine or haloperidol. Br J Psychiatry J Ment Sci. 2005 Dec;187:537–43.
24. Garriga M, Fernandez-Egea E, Mallorqui A, Serrano L, Oliveira C, Parellada E, et al. Antipsychotic-induced weight gain and birth weight in psychosis: A fetal programming model. J Psychiatr Res. 2019 Aug;115:29–35.
25. Lago SG, Tomasik J, van Rees GF, Rubey M, Gonzalez-Vioque E, Ramsey JM, et al. Exploring cellular markers of metabolic syndrome in peripheral blood mononuclear cells across the neuropsychiatric spectrum. Brain Behav Immun. 2021 Jan;91:673–82.
26. Zhou XM, Hu MR, Gong MY, Zou XL, Yu ZM. Sex-differential effects of olanzapine vs. aripiprazole on glucose and lipid metabolism in first-episode schizophrenia. Arch Clin Psychiatry São Paulo [Internet]. 2019 Apr [cited 2023 Jan 11];46(2):33–9. Available from: <http://www.scielo.br/scielo.php?script=sci_arttext&pid=S0101-60832019000200033&tlng=en>
27. Vázquez-Bourgon J, Mayoral-van Son J, Gómez-Revuelta M, Juncal-Ruiz M, Ortiz-García de la Foz V, Tordesillas-Gutiérrez D, et al. Treatment Discontinuation Impact on Long-Term (10-Year) Weight Gain and Lipid Metabolism in First-Episode Psychosis: Results From the PAFIP-10 Cohort. Int J Neuropsychopharmacol. 2021 Jan 20;24(1):1–7.

# Part 8 – Novel prognostic factors studied.

We identified several novel biomarkers including neurological assessments and biological biomarkers that were studied for their association with AIWG prognosis. Neurological assessments were of areas believed to be involved in hedonic appetite regulation. One study (n=69) tested the hypothesis that variable predisposition to AIWG may be mediated in part by varying levels of activity in the dorsal striatum during reward anticipation, resulting in a predisposition for abnormal craving behaviour and overeating. When adjusted for age and weight, lower baseline activity in the right-sided putamen was found to be associated with amisulpride-induced weight gain at six weeks, b= 0.20 (95% CI -0.02-0.42), p=0.06, suggesting a potential role of attenuated reward activity in the mesolimbic pathway in AIWG aetiology.^1^ Evidence quality supporting pre-antipsychotic reward anticipation assessments as a prognostic factor was however judged to be low. A second study (n=81) assessed striatal volume and connectivity with other areas believed to be involved in appetite regulation and reported that higher left putamen volume, β = 0.31 (95% CI 0.03-0.59), p=0.033

and lower connectivity with the lateral part of the right sensory motor cortex (numerical results not reported) correlated with magnitude of weight gain at 12 weeks antipsychotic treatment, when weight was measured in pounds (lbs).^2^ Evidence quality was judged to be low in the case of both striatal volume and connectivity assessments as potential prognostic factors. In another study (n=90) assessment of pre-antipsychotic hippocampal subfield volumes in predicting BMI change amongst those prescribed flupenthixol for 52 weeks was undertaken, given the role of the hippocampus in cognitive appetite control. Adjusted analysis revealed an association between anterior hippocampal volume and change in BMI, b = 3.03 (95% CI 0.11-5.95), p=0.046. Posterior hippocampal volume was associated with more imprecise results, b = 2.18 (95% CI =-1.50–5.86), p=0.250.^3^ Quality for this prognostic factor was also low. Prognostic factors requiring subjective interpretation e.g., imaging results, need extensive further examination due to risk of studying the predictive ability of the observer rather than that of the prognostic factor. Considering this, quality of supporting evidence and the absence of use of imaging results in current psychiatric diagnostic and prognostic evaluations, neurological assessments discussed here require further confirmatory studies to confirm the associations reported here to their practical prognostic value. Evidence quality supporting biomarkers was generally very low, except for peripheral metabolic markers and prolactin measurement assessed in single studies,^4,5^ although in both cases, even in the absence of serious quality defects, effect sizes reported thus far are not of significance to current clinical practice. In the case of antioxidant enzymes,^6^ pro-inflammatory cytokines,^7^ and various other routinely measured clinical parameters including urea and thyroxine measurements, significant concerns of bias seriously limited any conclusions that could be drawn from assessments.

***References***

1. Nielsen MØ, Rostrup E, Wulff S, Glenthøj B, Ebdrup BH. Striatal Reward Activity and Antipsychotic-Associated Weight Change in Patients With Schizophrenia Undergoing Initial Treatment. JAMA Psychiatry. 2016 Feb;73(2):121–8.
2. Homan P, Argyelan M, Fales CL, Barber AD, DeRosse P, Szeszko PR, et al. Striatal volume and functional connectivity correlate with weight gain in early-phase psychosis. Neuropsychopharmacol Off Publ Am Coll Neuropsychopharmacol. 2019 Oct;44(11):1948–54.
3. Luckhoff HK, du Plessis S, Kilian S, Asmal L, Scheffler F, Phahladira L, et al. Hippocampal subfield volumes and change in body mass over 12 months of treatment in first-episode schizophrenia spectrum disorders. Psychiatry Res Neuroimaging. 2020 Jun 30;300:111084.
4. Lago SG, Tomasik J, van Rees GF, Rubey M, Gonzalez-Vioque E, Ramsey JM, et al. Exploring cellular markers of metabolic syndrome in peripheral blood mononuclear cells across the neuropsychiatric spectrum. Brain Behav Immun. 2021 Jan;91:673–82.
5. Chiliza B, Asmal L, Oosthuizen P, van Niekerk E, Erasmus R, Kidd M, et al. Changes in body mass and metabolic profiles in patients with first-episode schizophrenia treated for 12 months with a first-generation antipsychotic. Eur Psychiatry J Assoc Eur Psychiatr. 2015 Feb;30(2):277–83.
6. Liu H, Yu R, Gao Y, Li X, Guan X, Thomas K, et al. Antioxidant enzymes and weight gain in drug-naive first episode schizophrenia patients treated with risperidone for 12 weeks: a prospective longitudinal study. Curr Neuropharmacol. 2021 Sep 19;
7. Song X, Fan X, Li X, Zhang W, Gao J, Zhao J, et al. Changes in pro-inflammatory cytokines and body weight during 6-month risperidone treatment in drug naïve, first-episode schizophrenia. Psychopharmacology (Berl). 2014 Jan;231(2):319–25.
8. Li S, Gao Y, Lv H, Zhang M, Wang L, Jiang R, et al. T4 and waist:hip ratio as biomarkers of antipsychotic-induced weight gain in Han Chinese inpatients with schizophrenia. Psychoneuroendocrinology. 2018 Feb;88:54–60
